# Novel plant-derived exosome-like nanovesicles from *Catharanthus roseus*: preparation, characterization, and immunostimulatory effect via TNF-α/NF-κB/PU.1 axis

**DOI:** 10.1186/s12951-023-01919-x

**Published:** 2023-05-20

**Authors:** Xiaozheng Ou, Haoran Wang, Huilin Tie, Jiapei Liao, Yuanyuan Luo, Weijuan Huang, Rongmin Yu, Liyan Song, Jianhua Zhu

**Affiliations:** 1 Biotechnological Institute of Chinese Materia Medica, Jinan University, Guangzhou, 511443 China; 2 Department of Pharmacology, Jinan University, Guangzhou, 511443 China; 3 Weihai Neoland Biosciences Co., Ltd, Weihai, 264209 China

**Keywords:** Plant-derived exosome-like nanovesicles, *Catharanthus roseus* (L.) Don, Immunostimulation, Plant cell culture technology

## Abstract

**Background:**

Plant-derived exosomes-like nanovesicles (PDENs) have been found to be advantageous in disease treatment and drug delivery, but research on their biogenesis, compositional analysis, and key marker proteins is still in its infancy, which limits the standardized production of PDENs. Efficient preparation of PDENs continues to be a major challenge.

**Results:**

Novel PDENs-based chemotherapeutic immune modulators, *Catharanthus roseus* (L.) Don leaves-derived exosome-like nanovesicles (CLDENs) were isolated from apoplastic fluid. CLDENs were membrane structured vesicles with a particle size of 75.51 ± 10.19 nm and a surface charge of −21.8 mV. CLDENs exhibited excellent stability, tolerating multiple enzymatic digestions, resisting extreme pH environments, and remaining stable in the gastrointestinal simulating fluid. Biodistribution experiments showed that CLDENs could be internalized by immune cells, and targeted at immune organs after intraperitoneal injection. The lipidomic analysis revealed CLDENs’ special lipid composition, which contained 36.5% ether-phospholipids. Differential proteomics supported the origin of CLDENs in multivesicular bodies, and six marker proteins of CLDENs were identified for the first time. 60 ~ 240 μg/ml of CLDENs promoted the polarization and phagocytosis of macrophages as well as lymphocyte proliferation in vitro. Administration of 20 mg/kg and 60 mg/kg of CLDENs alleviated white blood cell reduction and bone marrow cell cycle arrest in immunosuppressive mice induced by cyclophosphamide. CLDENs strongly stimulated the secretion of TNF-α, activated NF-κB signal pathway and increased the expression of the hematopoietic function-related transcription factor PU.1 both in vitro and in vivo. To ensure a steady supply of CLDENs, plant cell culture systems of *C. roseus* were established to provide CLDENs-like nanovesicles which had similar physical properties and biological activities. Gram-level nanovesicles were successfully obtained from the culture medium, and the yield was three times as high as the original.

**Conclusions:**

Our research supports the use of CLDENs as a nano-biomaterial with excellent stability and biocompatibility, and for post-chemotherapy immune adjuvant therapy applications.

**Graphical Abstract:**

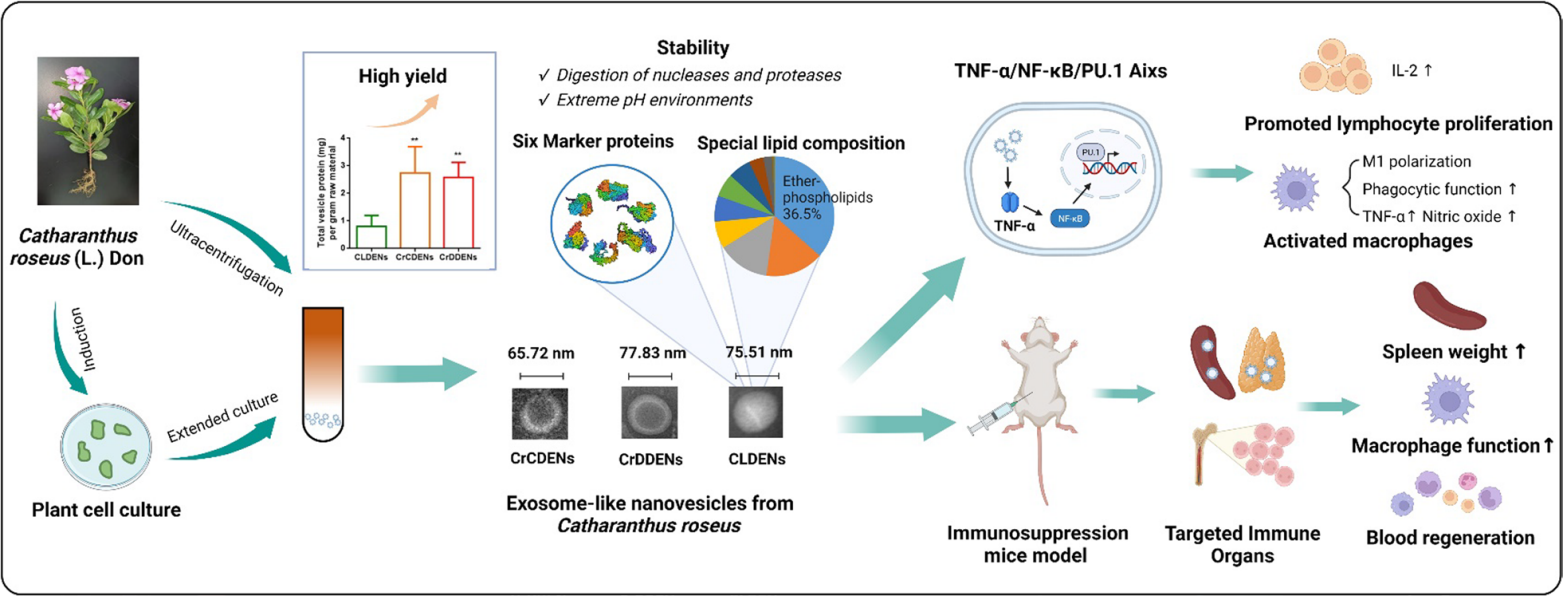

**Supplementary Information:**

The online version contains supplementary material available at 10.1186/s12951-023-01919-x.

## Background

Plant-derived exosomes-like nanovesicles (PDENs) are a class of natural active substances that have been widely reported in recent years. They have similar structure and function to mammalian exosomes [1] and have shown good efficacy in diseases such as cancer [2], inflammation as well as COVID-19 [3]. It has been discovered that PDENs can activate cross-kingdom regulation and transmit molecular signals between plant cells, bacteria, parasites, fungi [4], and even animals [5, 6], which demonstrated promising applications in the field of disease treatment.

As natural intercellular communication tools, PDENs and exosomes are also considered to have great potential for drug delivery. Compared to artificial nanoparticles, they have the advantages of natural molecular transport properties, lower immunogenicity and toxic side effects [7]. Researchers have successfully targeted brain diseases using exosome-loaded RNAi [8] and anti-cancer compounds loaded onto grapefruit-derived nanovesicles [9], which demonstrated their advantages of crossing biological barriers. However, it is reported that the drug delivery strategy of animal exosomes may cause potential biohazards such as stimulation of tumor proliferation [10]. Compared with animal exosomes, PDENs are not only less potentially biohazardous but also more economically feasible as drug carriers.

However, as the understanding of PDENs is still in the preliminary stage, the pharmacological application of PDENs faces many challenges. Only a few studies related to plant physiology have reported the formation mechanism of nanovesicles in model plants [11, 12]. For most plants, the biological mechanism of PDENs remains unknown. Further, methods to optimize the extraction process and improve the extraction efficiency of PDENs are also still being explored. Ultracentrifugation of plant juices continues to be the main way to isolate PDENs and showed low extraction efficiency [13]. Intact plants contain a large subpopulation of nanovesicles from different tissue sites, which may have different structures and functions. PDENs originating from different species have even more diverse compositions [14, 15] and biodistribution characteristics [2, 16]. This high degree of heterogeneity makes it very difficult to obtain reproducible generic or specific markers for PDENs, and limits the quality control as well as further industrial production of PDENs. Therefore, a systematic study of the composition, stability, biocompatibility, biodistribution characteristics and other important pharmacological parameters of PDENs is necessary. Although research on PDENs is not yet comprehensive enough, PDENs are considered to be a biological nanomaterial with great potential for development because of their good biocompatibility as well as the advantages of a wide source, a low price, and easy access.

Immunosuppression is one of the most common side effects of tumor chemotherapy, which often leads to the termination of chemotherapy and affects the effective treatment of tumors. Cyclophosphamide, a widely used chemotherapeutic agent can induce strong myelosuppression symptoms. High doses of cyclophosphamide can even increase the risk of infectious and hemorrhagic complications [17, 18]. Currently, colony-stimulating factor (CSF) is the prevailing solution in clinical practice to stimulate the differentiation of hematopoietic stem cells (HSC) to supplement the immune cell bank. However, CSF has been reported to have the risk of stimulating tumor growth [19]. Therefore, it is necessary to develop a safe immune modulator for chemotherapy.

It has been widely established that PDENs affect immune responses and inflammatory processes. They have an impact on the immune system by altering macrophage polarization [2], delivering miRNA [3], modifying the tumor microenvironment [20], and reducing inflammation [21]. A variety of plants and their extracts have been reported to play a unique role in relieving chemotherapy-induced immunosuppression [22]. However, the research on PDENs' effects on immunosuppression and hematopoietic functioning has not yet been extensively documented. Therefore, we sought to examine the feasibility of developing a novel chemotherapeutic immune modulator from PDENs.

*Catharanthus roseus* (L.) Don is a widely planted herb around the world with a long history of medicinal use [23]. Modern pharmacological research shows that there are many compounds in *C. roseus*, such as indole alkaloids, flavonoids, phenolic acids, polysaccharides, which can be used for anti-tumor [24], anthelmintic [25] and anti-diabetes [26]. According to Chinese traditional medicine literature, fresh *C. roseus* leaves smashed and applied to wounds can treat burns, scalds, canker sores and swelling sores [27]. *C. roseus* leaves and their extracts have been reported to resist microbial infections [28] and parasitic infestations [29], promote wound healing [30]. To cope with infection and wound healing, many immune cells (especially macrophages and neutrophils) need to be mobilized quickly. This processing requires the rapid response of HSC to continuously supplement the innate immune cell bank [31]. *C. roseus* may have the potential to regulate immunity and respond to acute hematopoietic mobilization. But the detailed mechanisms of these effects still remain unknown up to now.

In this study, we reported a novel PDENs with excellent stability and good biocompatibility, *C. roseus* leaves-derived exosome-like nanovesicles (CLDENs). CLDENs were able to be taken up by immune cells, target immune organs, and exert cross-species immunostimulatory effects in animals. We revealed the biogenesis, component composition, stability, biocompatibility, and biodistribution characteristics of CLDENs, and showed their immunomodulatory activity via TNF-α/NF-κB/PU.1 axis. Our study also demonstrated the feasibility of applying plant cell culture technology to the large-scale production of PDENs.

## Results

### Isolation of exosome-like nanovesicles from *C. roseus*

*C. roseus*-derived nanovesicles were separated by differential ultracentrifugation after being crushed and juiced from fresh *C. roseus*. It was found that *C. roseus*-derived nanovesicles contained three nanovesicles with different particle sizes according to experimental results from transmission electron microscopy (TEM) and dynamic light scattering (DLS) analysis (Additional file 1: Fig. S1B and C). To avoid possible interference between different plant tissues, nanovesicles were recovered from leaves (Additional file 1: Fig. S1D), stems (Additional file 1: Fig. S1E) and flowers (Additional file 1: Fig. S1F). These nanovesicles differ in morphology and size. Among them, leaves-derived nanovesicles with more homogeneous morphology and exosome-like characteristics were selected for the study.

**Fig. 1:**
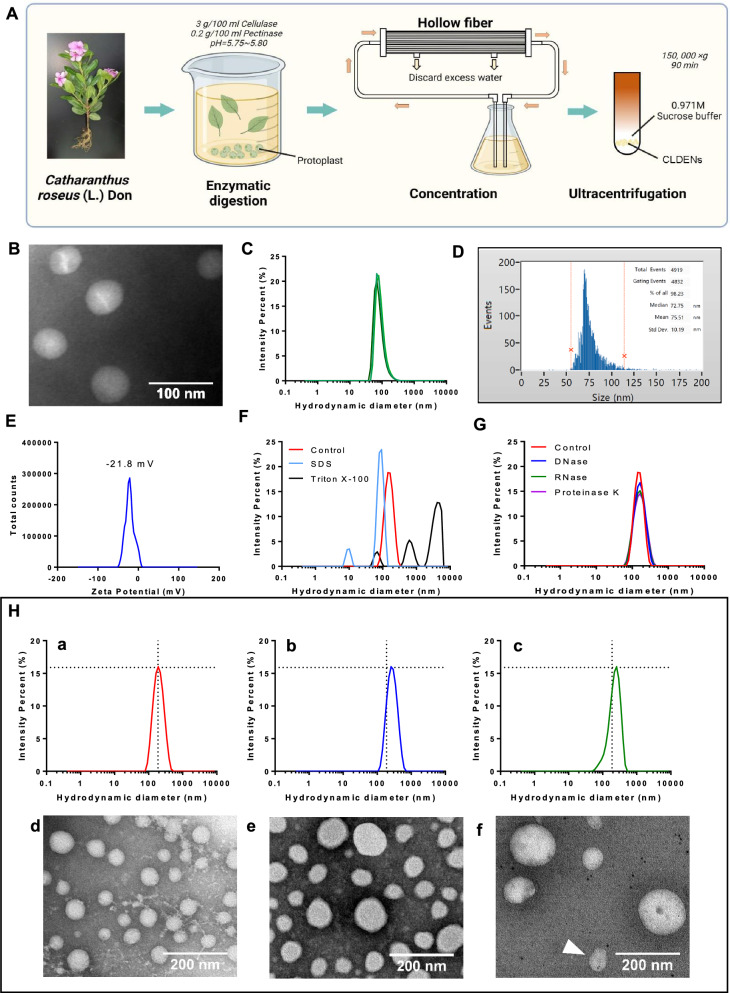
Isolation and physicochemical characterization of CLDENs. **A** Schematic diagram of the separation process of CLDENs. **B** TEM analysis of CLDENs. Scale bar = 100 nm. **C** DLS was used to analyze the particle size distribution of CLDENs. **D** A nanoflow analyzer picked up the CLDENs particle size. **E** Analysis of CLDENs’ zeta surface charge. **F**,**G** Particle size changes of CLDENs in enzyme (**F**) and surfactant-containing (**G**) environments. **H** Stability of CLDENs in different pH environments. Particle size changes of CLDENs in pH = 6.92 (a), pH = 13.58 (b) and pH = 1.83 (c) environments. TEM imagines of CLDENs in pH = 6.92 (d), pH = 13.58 (e) and pH = 1.83 (f) environments. Scale bar = 200 nm. The white triangle marked the two fused vesicles

We speculated that the juicing process can cause the plant to release large amounts of water-soluble pigments and also damaged the plant cells making the organelles spill out (especially chloroplasts, etc.). In order to obtain more purified extracellular vesicles, the extraction method was modified according to Ref [32] (Additional file 1: Fig. S1G and H). In brief, the leaves of *C. roseus* were cleaned, digested with pectinase and cellulase, and then centrifuged to remove protoplasts. The supernatant was concentrated using hollow fibers, and then CLDENs were extracted using sucrose buffer-ultracentrifugation (Fig. 1A). Extracellular vesicles isolated using the aforementioned technique were suspended in phosphate buffer solution and kept at −80 °C until use.

### Physicochemical characterization of CLDENs

Particle size analysis on CLDENs was tested by TEM within 48 h after the extraction. It was demonstrated that CLDENs had a rounded hollow vesicle shape and had particle size between 50 and 100 nm (Fig. 1B). DLS analysis indicated that the particle size of CLDENs in aqueous solution was 141.7 nm (Fig. 1C). We noticed that the difference between DLS analysis and TEM analysis in the detection of exosome particle size was a common phenomenon [33]. Therefore, we additionally used nano-flowmeter to complement the particle size of CLDENs and found that the particle size of CLDENs were 75.51 ± 10.19 nm (Fig. 1D).

Zeta potential is a significant characteristic that can be used to assess the stability of nanoparticle colloids. In general, nanoparticles with zeta potential values between -30 mV ~ + 30 mV are considered to have better stability [34]. It was found that CLDENs had a zeta potential of -21.8 mV in aqueous solution, indicating that the vesicles were stable (Fig. 1E).

Multiple methodologies were used to evaluate CLDENs’ stability. CLDENs were exposed to 0.02% Triton X-100 and 0.1% SDS for 30 min. The original single particle size peak of CLDENs split, indicating the destruction of vesicles (Fig. 1F). After being digested for 30 min with RNase (3 μg/ml), DNase (6 μg/ml), and proteinase K (100 μg/ml), the particle size distribution of CLDENs did not alter significantly, which indicated that the vesicles did not suffer damage (Fig. 1G). When immersed in sodium hydroxide solution and hydrochloric acid solution for 30 min, the particle size peak of CLDENs was not split but displaced (Fig. 1Ha-c). The variation of biomaterials in different pH environments is an important method to evaluate their stability [35]. To determine whether the rupture of CLDENs occurred, we observed the morphology of CLDENs under TEM after acid or alkali treatment. Compared with the control group (Fig. 1Hd), some nanovesicles expanded in size in an alkaline environment (Fig. 1He). This phenomenon was more obvious in an acidic environment, and the volume of vesicles changed from about 70 nm to more than 200 nm (Fig. 1Hf). As an acidic organelle, the change of pH environment can affect the membrane exchange and membrane fusion of MVB [36, 37]. Interestingly, we also observed the vesicle fusion of CLDENs in an acidic environment (Fig. 1Hf, Additional file 1: Fig. S2A and B). Even though, CLDENs still maintained a membrane vesicle-like structure without significant rupture even in acidic or alkaline environments. These data indicated that surfactants were able to break down CLDENs. CLDENs were resistant to acidic and alkaline environments, as well as the digestion of DNase, RNase, and protease.

### Biocompatibility and biodistribution of CLDENs

**Fig. 2:**
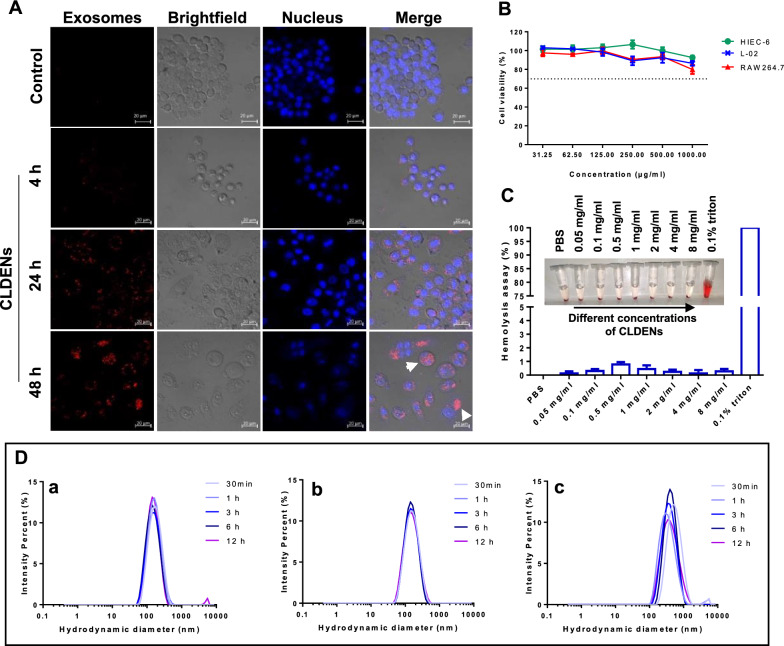
Biocompatibility of CLDENs. **A** CLDENs accumulated intracellularly over time after uptake. White triangles were used to mark RAW264.7 cells’ morphological changes after ingestion of CLDENs. Scale bar = 20 μm. **B** Cytotoxicity assay of CLDENs against multiple cell lines (n = 3). **C** Hemolysis test under different concentrations of CLDENs by using blood of sheep (n = 3). **D** Changes in the particle size distribution of CLDENs after incubation in PBS (a), stomach simulating fluid (b) and small intestine simulating fluid (c) at 37 °C

CLDENs were labeled with red fluorescent dye PKH26 and were co-incubated with RAW 264.7 cells. It was showed that CLDENs were taken up by macrophages and deposited primarily in the cell cytoplasm. The red fluorescent signal gradually increased in RAW264.7 cells by time prolonged (Fig. 2A). Additionally, as the co-incubation period increased, the morphology of the RAW264.7 cells gradually changed, primarily as evidenced by an increase in cell volume and the number of pseudopodia (Marked by the white triangle in Fig. 2A).

The MTT assay was used for detecting CLDENs’ cytotoxic effects on HIEC-6 cells, L-02 cells, RAW264.7 cells. Based on well-established guidelines ISO 10993.5, the materials for medical use were considered not cytotoxic when the cell viability rate was greater than 70% [38]. It appeared that the cell viability rate remained above 70% even at the highest concentration (1000 μg/ml) of CLDENs (Fig. 2B). These data suggested that CLDENs were relatively safe nano-vesicles.

Whole sheep blood was used for the evaluation of the hemolytic effect of CLDENs. As shown in Fig. 2C, no significant hemolysis was observed after incubating sheep blood with 0.05–8 mg/mL of CLDENs at 37 °C for 30 min. Compared with the negative control (PBS, which was regarded as having a 0% hemolysis rate) and the positive control (0.1% Triton X-100, which was regarded as having a 100% hemolysis rate), the hemolysis rate of CLDENs were less than 1%. These data showed that CLDENs possessed good biocompatibility.

Before observing the in vivo distribution of CLDENs, we examined the stability of CLDENs in an artificially simulated in vivo environment. The particle size distribution of CLDENs will not be affected when it was placed at 37 ℃ for 12 h (Fig. 2Da). Moreover, when CLDENs were placed in small intestine simulating fluid (Fig. 2Db) and stomach simulating fluid (Fig. 2Dc) at 37 °C, they could maintain stability for at least 12 h.

Then, we gave mice injections in the tail vein, intraperitoneal administration, and oral administration of 60 mg/kg of CLDENs, which were labeled with the near-infrared lipophilic fluorescent dye DIR. At regular intervals, animals were killed and their organs and tissues were taken to look for fluorescent signals.

**Fig. 3:**
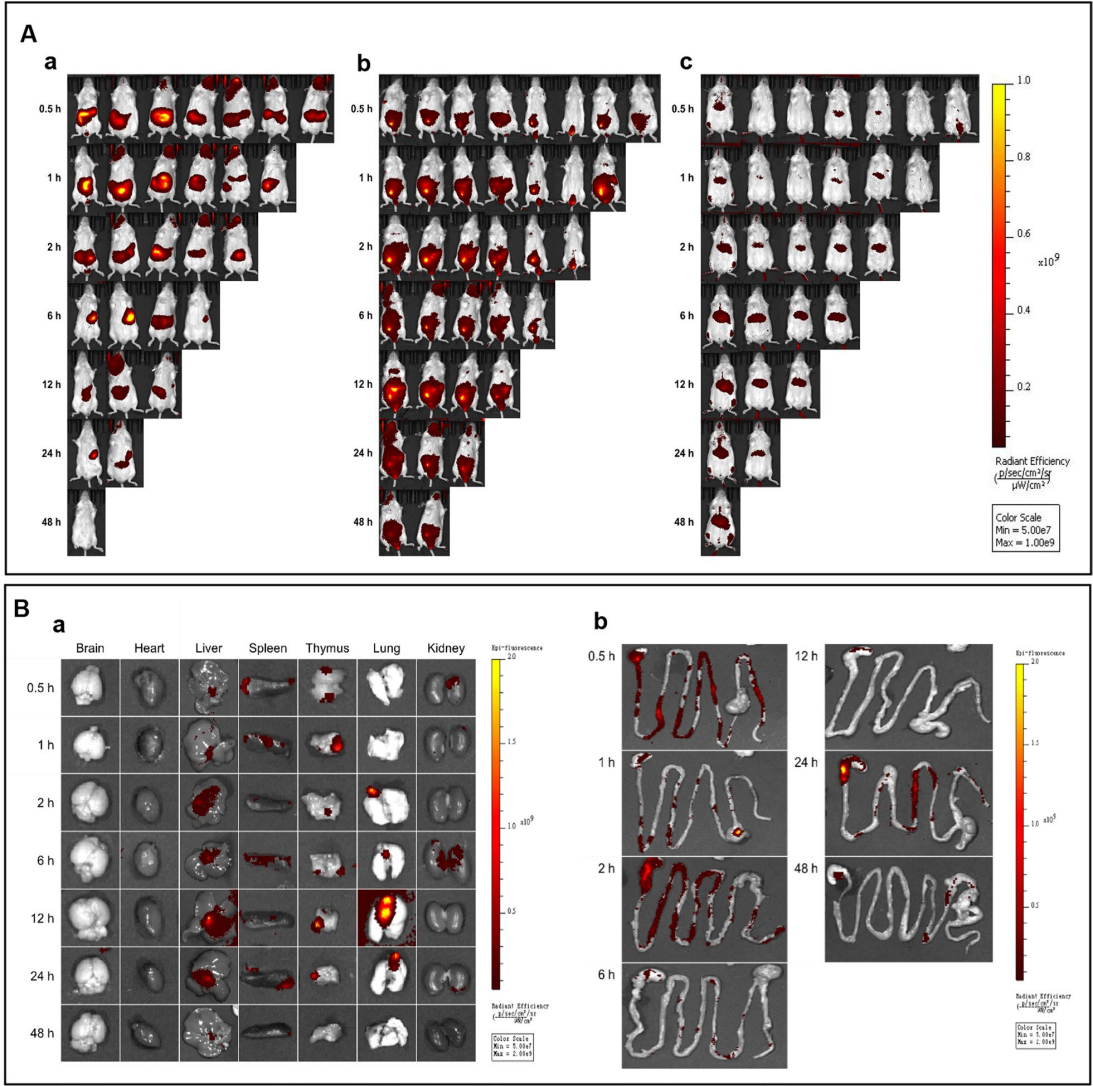
Biodistribution of CLDENs. **A** Biodistribution of CLDENs in mice after oral administration (a), intraperitoneal injection (b), and tail vein injection (c). **B** Biodistribution of CLDENs in the organs after intraperitoneal injection administration. a. Fluorescent signals in the brain, heart, liver, spleen, thymus, lung and kidney. b. Fluorescent signals in the gastrointestinal tract

Following oral treatment, only in the mice's abdominal region, the signs of CLDENs-DIR seen (Fig. 3Aa). After the animals were dissected, a minor fluorescent signal was seen in the liver, brain and lung (Additional file 1: Fig. S3Aa). Besides, a sustained, intense fluorescent signal was discovered in the mouse stomach which was above the instrument detection threshold and gradually waned after 12 h (Additional file 1: Fig. S3Ab). The CLDENs-DIR fluorescent signal did not expel from the stomach, even though the mice had free access to food and water. Combined with the results of in vitro experiments, we hypothesized that CLDENs may remain stable in gastric juice and have potent stomach absorption.

In the intraperitoneal injection group, CLDENs were found to be distributed in the immune organs. Fluorescent signals were observed in the thymus, spleen, pulmonary vasculature, liver, kidney (Fig. 3Ba) and the gastrointestinal system (Fig. 3Bb). In animals’ bodies, the fluorescent signal first appeared in the abdomen, and then in the testes, paws, and spread to the head over time (Fig. 3Ab), although no fluorescent signal was observed in the brain. It's interesting to note that some animals’ cervical lymphatic fraction also showed CLDENs-DIR signals (Additional file 1: Fig. S4).

Consistent with earlier reports, tail vein injection did not show more extensive systemic absorption than intraperitoneal injection [39]. CLDENs-DIR were primarily found in the animal's upper abdomen and extremities, except for the caudal injection site (Fig. 3Ac). Moreover, the tail vein injection group showed a much weaker fluorescent signal at the same scale. Only the liver and spleen of the animals' dissected organs contained CLDENs-DIR (Additional file 1: Fig. S3Ba). We hypothesized that CLDENs-DIR were quickly captured and processed by the liver and spleen after entering the circulatory system. Furthermore, robust fluorescent signals in the liver continued to be seen for 48 h.

To sum up, different modes of administration can cause CLDENs to exhibit very different biodistributions. Our data suggested that CLDENs may have excellent anti-gastric acid digestibility and can remain in the gastrointestinal tract for a long time after oral administration. Moreover, immune organ-targeted distribution can occur by intraperitoneal injection of CLDENs. CLDENs injected intravenously into the bloodstream may face interception and rapid clearance by the liver and spleen.

### Compositional analysis of CLDENs

**Fig. 4:**
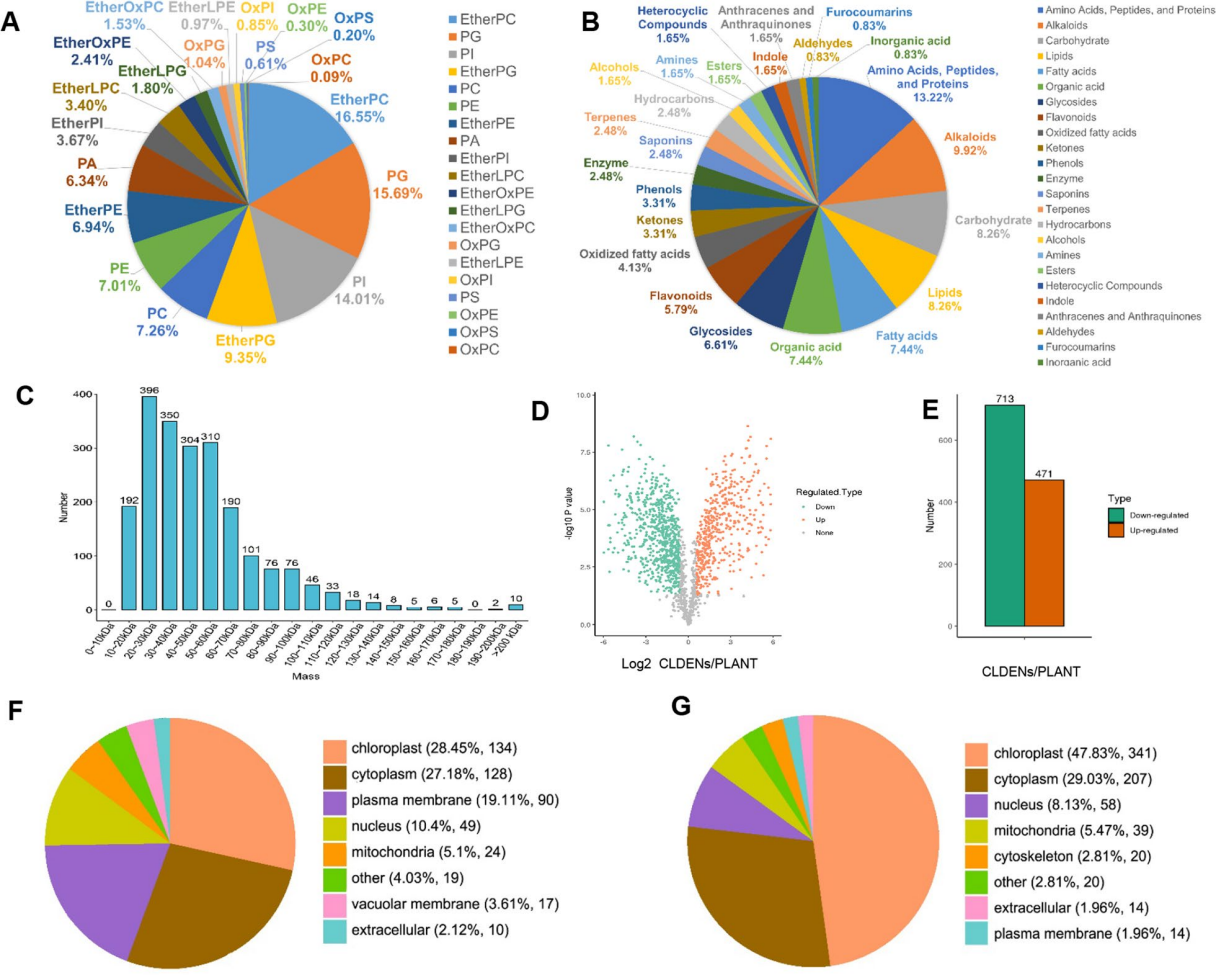
Compositional analysis of CLDENs. **A** Lipidomic analysis of CLDENs. **B** Metabolomic analysis of CLDENs. **C** Molecular weights distribution of the identified proteins in the CLDENs group. **D** Volcano plot of proteomic analysis. **E** The number of differentially up-regulated and down-regulated expressed proteins. **F**, **G** Subcellular localization of differentially expressed proteins, up-regulated proteins (**F**) and down-regulated proteins (**G**)

As a biological membrane vesicle, lipid makes up the majority of extracellular vesicles [40]. Non-targeted lipidomic was used to analyze the lipid composition of CLDENs. It was found that CLDENs contained mainly ether-phosphatidylcholines (EtherPC, ~ 16.55%), phosphatidylglycerols (PG, ~ 15.69%), phosphatidylinositol (PI, ~ 14.01%) and ether-phosphatidylglycerols (EtherPG, ~ 9.35%) (Fig. 4A and Additional file 1: Table S1). Exosomes are usually rich in saturated phospholipids, sphingomyelin, cholesterol, and phosphatidylserine [33, 41]. It was worth noting that CLDENs contained over 30% ether-phospholipids. Ether-phospholipids are a distinct type of phospholipid in which the sn-1 arm of the glycerol backbone is linked by an ether bond rather than an ester bond [42]. They play an important role in promoting the differentiation of neural cells [43], and the activation of neutrophils and macrophages [44, 45]. Moreover, ether-phospholipids have been reported to maintain membrane structure and integrity in nematode-derived exosomes, and participate in the host immune response [46]. The high content of ether-phospholipids may be responsible for the excellent stability exhibited by CLDENs. Also, these findings prompted us to speculate about CLDENs’ potential involvement in the immunomodulatory processes.

Non-targeted metabolomic analysis was performed to identify compounds contained in CLDENs. The result showed that CLDENs mainly contained compounds such as amino acids, fatty acids and lipids, alkaloids, and carbohydrates (Fig. 4B and Additional file 1: Table S2). We paid special attention to the outstanding antitumor compounds contained in *C. roseus,* indole alkaloids. Vinpocetine was found in CLDENs, but not the well-known vinblastine, vincristine, amorphine, or vinblastine. It was reported that secondary metabolite enrichment was not exhibited all PDENs. Researchers found nanovesicles derived from grapefruit and ginger included naringin, naringenin [16], and shogaol [47], whereas those derived from orange lacked vitamin C and naringenin [48]. In this regard, we hypothesized that the types of compounds present in PDENs were influenced by the extraction environment at various pH values and the lipophilic characteristics of the compounds [49]. A disease correlation analysis of the identified compounds was performed based on the BATMAN-TCM online analysis tool. We examined which disease phenotypes CLDENs might be used to treat based on the disease targets information of identified compounds. The results indicated that CLDENs might have therapeutic potential for multiple severe combined immunodeficiency diseases and human immunodeficiency virus (Additional file 1: Table S3). These results demonstrated the possibility of applying CLDENs to immune system diseases.

Then, we performed differential proteomic analysis of *C. roseus* leaves and CLDENs. 1843 proteins were identified and quantified in the CLDENs group (Fig. 4C), compared to 3241 in the PLANT group (Additional file 1: Fig. S5). In CLDENs group, 86.04% of the identified proteins had molecular weights distributed between 10 and 80 kDa. After comparing the PLANT group and CLDENs group, a total of 1184 differentially expressed proteins were identified, of which 471 were significantly up-regulated and 713 were significantly down-regulated (Fig. 4D and E). Both up-regulated proteins and down-regulated proteins showed a clear tendency to localize to the chloroplast (Up: 28.45%, Down: 47.83%) and cytoplasm (Up: 27.18%, Down: 29.03%). But the up-regulated proteins had more proteins localized to the plasma membrane (Up: 19.11%, Down: 1.96%) (Fig. 4F and G).

Analysis around various up-regulated proteins revealed that CLDENs had an enrichment of transport and signaling proteins such as ABC transporter family members, enzymes like pyruvate decarboxylase, and endosomal membrane proteins represented by DnaJ homolog subfamily C GRV2-like protein. The ratio of relative quantification of significantly up-regulated proteins in *C. roseus* leaves and CLDENs were examined. The CLDENs/PLANT Ratio values of the top six up-regulated proteins were all greater than 100 (Additional file 1: Table S4), indicating that they were significantly enriched in CLDENs and can be thought of as the marker proteins of CLDENs. They were pyruvate decarboxylase, ABC transporter C family member 10, probable metal-nicotianamine transporter YSL6 isoform X1, glycerophosphodiester phosphodiesterase, DnaJ homolog subfamily C GRV2-like, and ABC transporter C family member 4-like.

Among these proteins significantly enriched in CLDENs (Additional file 1: Table S4), we found proteins associated with multivesicular and intraluminal vesicles, such as DnaJ homolog subfamily C GRV2-like protein, AP-3 complex subunit delta [50], Flotillin-like protein [51]. Particularly the DnaJ homolog subfamily C GRV2-like protein, a crucial protein in *Arabidopsis thaliana*'s late endosome development. It is localized in the endosomal membrane and involved in multivesicular mediated vesicle trafficking [52]. Exosomes are thought to be tiny vesicles that are released outside the cell after binding to the plasma membrane by MVB [53]. The enrichment of these proteins provided evidence of CLDENs’ origin from MVB.

### Immunostimulatory effects of CLDENs via TNF-α/ NF- κ B/PU.1 axis

**Fig. 5:**
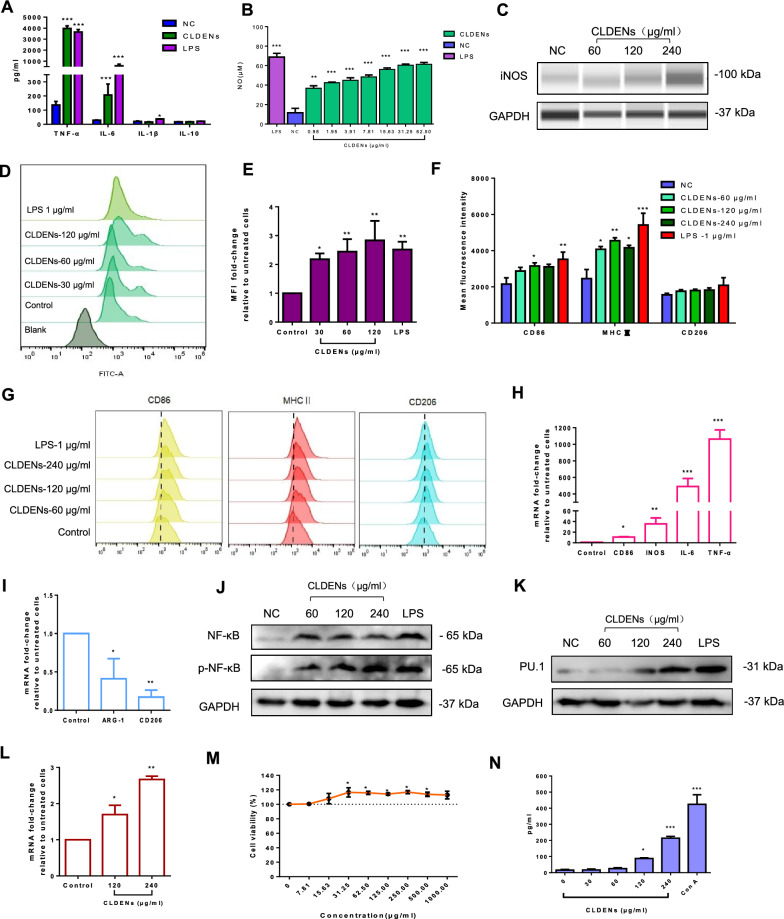
Immunostimulatory effects of CLDENs in vitro. **A** CLDENs promoted the secretion of cytokines TNF-α and IL-6 from RAW264.7 cells, while it had no significant promotion effect on the secretion of IL-1β and IL-10. **B** CLDENs promoted nitric oxide release from RAW264.7 cells. **C** CLDENs increased the expression level of nitric oxide synthase in RAW264.7 cells. **D**, **E** Effect of CLDENs on the ability of RAW264.7 cells to phagocytose FITC-Dextran. Data were shown in mean fluorescence intensity (**D**) and histogram (**E**). **F**, **G** CLDENs enhanced RAW macrophage surface antigen CD86 and MHC II expression but had no discernible impact on CD206 expression. Data were shown in mean fluorescence intensity (**G**) and histogram (**F**). **H** CLDENs increased the mRNA transcription levels of M1-type related genes in RAW264.7 cells. **I** CLDENs reduced the mRNA transcription levels of M2-type related genes in RAW264.7 cells. **J** CLDENs activated the transduction of the NF-κB signaling pathway. **K** CLDENs up-regulated the expression level of PU.1 protein in RAW264.7 cells. **L** CLDENs up-regulated the mRNA transcript level of PU.1 gene in RAW264.7 cells. **M** Effect of CLDENs on the cell viability of primary spleen lymphocytes. **N** CLDENs promoted the secretion of IL-2 from primary splenic lymphocytes. Data were mean ± SD, n = 3; ^*^P < 0.05, ^**^P < 0.01 and ^***^P < 0.001 *vs.* Control

Subsequently, we examined whether CLDENs played a role in inflammatory and immune processes. The secretion of cytokines could directly reflect the involvement of immune cells in the immune response [54]. It was found that CLDENs greatly stimulated the secretion of TNF-α (Fig. 5A). High levels of TNF-α were found in the RAW264.7 cells’ culture supernatant after 24 h of co-incubation with 120 g/ml CLDENs (3975.74 ± 469.24 pg/ml), which was over 30 times greater than the normal control group (135.96 ± 50.56 pg/ml) and exceeded the lipopolysaccharide (LPS) group (3650.09 ± 442.43 pg/ml). Besides, CLDENs dramatically increased the secretion of interleukin-6 (IL-6) by RAW264.7 cells and showed no discernible impact on interleukin-1β (IL-1β) or interleukin-10 (IL-10). In the concentration range of 0.98 ~ 62.5 μg/ml, CLDENs significantly stimulated RAW264.7 cells to secrete nitric oxide (Fig. 5B). From the protein level, an up-regulation of inducible nitric oxide synthase (iNOS) expression level was observed in RAW264.7 cells after CLDENs treatment (Fig. 5C). These results indicated that CLDENs promoted macrophages to secrete a variety of cytokines and activate the inflammatory response.

In addition, in order to detect the relationship between the structural integrity and activity of CLDENs, they were treated with enzymes, acids, bases, and surfactants before expose to RAW264.7 cells. The results showed that neither the acid–base treatment nor the 30-min enzymatic digestion treatment affected CLDENs’ ability to stimulate nitric oxide release from RAW264.7 cells. Similar to the previous results, this effect could be completely eliminated by surfactant and heat treatment (Additional file 1: Fig. S6). The above results indicating that structural integrity of CLDENs was essential for its immunomodulatory activity.

A FITC-Dextran fluorescent particle uptake experiment was used to detect the phagocytic function of macrophages. The results showed that CLDENs significantly enhanced the phagocytic function of RAW264.7 cells (Fig. 5D). The mean fluorescence intensity in 120 μg/ml CLDENs group was three times higher than that in the control group (Fig. 5E).

Macrophages usually differentiate into two major phenotypes M1 and M2 after being activated, which indicates the progression of the inflammatory response. Positive expression of macrophage surface antigen CD86 and MHC II is usually considered as M1-type macrophage, while CD206 is considered as M2-type macrophage [55, 56]. Flow cytometry was used to detect the influence of CLDENs on the expression of macrophage surface antigens on RAW264.7 cells. The results showed that CLDENs significantly increased the expression of CD86 and MHC II (Fig. 5F and G) though CD206 was unaffected by CLDENs. After 24 h of incubation with 120 μg/ml CLDENs, compared to the normal control group, RAW264.7 cells had significantly higher levels of mRNA transcription for M1 macrophage-related genes CD86, iNOS, IL-6, and TNF-α (Fig. 5H), but significantly lower levels for M2 macrophage-related genes Arginase-1 and CD206 (Fig. 5I). CLDENs greatly promoted the differentiation of RAW264.7 cells and induced their polarization toward the M1 type.

The above data demonstrated that CLDENs strongly stimulated macrophages to secrete TNF-α, enhanced phagocytosis of macrophages as well as promoted immune response-related cell differentiation. TNF-α can activate inflammation-related signaling pathways represented by the NF-κB pathway and induces downstream immunomodulation-related cascade responses [57]. Among which PU.1 is a key transcription factor in the regulation of immune cell growth, development, and function [58, 59]. Therefore, western blot assay was performed to detect the protein express level of NF-κB and PU.1. As expected, when RAW264.7 cells were treated CLDENs for 24 h, the expression and phosphorylation of NF-κB were increased (Fig. 5J, LPS as positive control). Also, CLDENs significantly increased the expression of PU.1 in RAW264.7 cells, both at the protein level (Fig. 5K, LPS as positive control) and at the transcriptional level (Fig. 5L). The above data demonstrated that CLDENs strongly stimulated macrophages to secrete TNF-α, enhanced phagocytosis of macrophages as well as promoted immune response-related cell differentiation.

Comparable effects were observed in splenic lymphocytes. Spleen lymphocytes were separated from mouse spleens and co-incubated with CLDENs for 24 h. CLDENs significantly promoted spleen lymphocyte proliferation (Fig. 5M). ELISA experiment results showed that CLDENs substantially improve splenic cells' release of interleukin-2 (IL-2) (Fig. 5N).

In short, CLDENs promoted the proliferation and differentiation of immune cells and facilitated their functional activation in vitro. This immunomodulatory effect was mediated through the TNF-α/NF-κB/PU.1 axis. Additionally, the intact vesicle structure and suitable temperature were required for CLDENs to function.

### CLDENs alleviated cyclophosphamide-induced immunosuppression

**Fig. 6:**
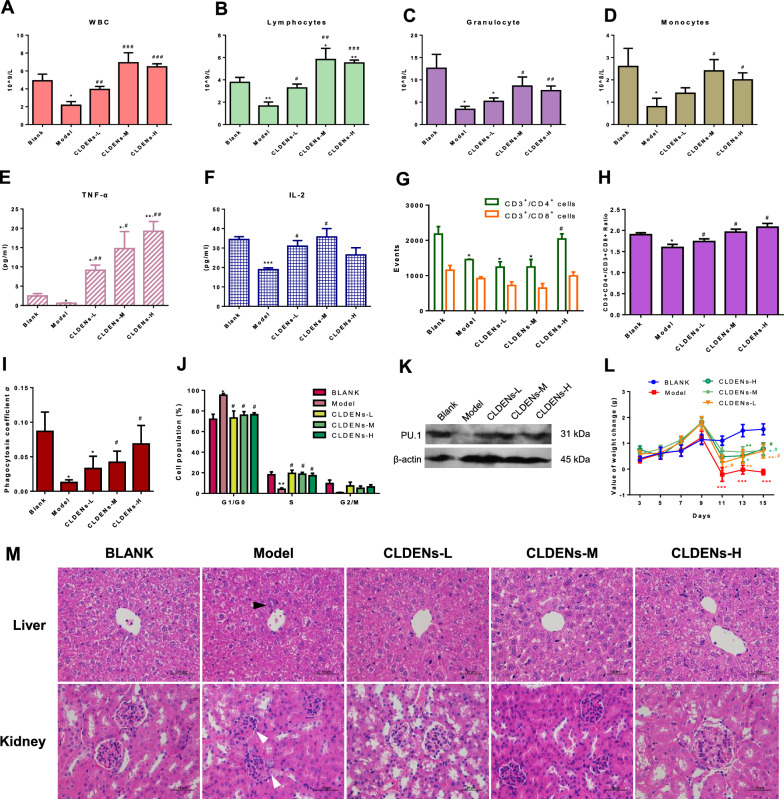
CLDENs alleviated cyclophosphamide-induced immunosuppression. **A**–**D** Effect of CLDENs on the number of white blood cells (**A**), lymphocytes (**B**), neutrophils (**C**), and monocytes (**D**) in the peripheral blood of immunosuppressed mice (n = 5). **E**, **F** Effect of CLDENs on the secretion levels of cytokines TNF-α (**E**) and IL-2 (**F**) in the serum of mice in a cyclophosphamide immunosuppression model (n = 3). **G**, **H** Effect of CLDENs on splenic lymphocyte subpopulations of immunosuppressed mice, number (**G**) and proportion (**H**). **I** Impact of CLDENs on the immunosuppressed mice's ability to remove carbon particles (n = 3) **J** Effects of CLDENs on cell cycle distribution of bone marrow cells in immunosuppressed mice (n = 3). **K** Effect of CLDENs on the relative body weight gain of cyclophosphamide-induced immunosuppressed mice (n = 10). **L** Effects of CLDENs on the liver and kidney tissues of cyclophosphamide-induced immunosuppression mice were analyzed by hematoxylin–eosin staining. Data were mean ± SD; ^*^P < 0.05, ^**^P < 0.01 and ^***^P < 0.001 vs. Blank; ^#^*P* < 0.05, ^##^*P* < 0.01 and ^###^*P* < 0.001 vs. Model

An immunosuppressed mouse model was established by continuous 3-day administration of 80 mg/kg cyclophosphamide. In order to observe the possible toxic effects of CLDENs, we set the dose at a concentration gradient of nearly 3 times, that was 6 mg/kg, 20 mg/kg and 60 mg/kg. According to reports, the number of blood cell populations in peripheral blood can be used clinically to determine whether a patient is experiencing myelosuppression [60]. The routine blood tests showed that cyclophosphamide significantly reduced the number of white blood cells, lymphocytes, granulocytes, and monocytes in the peripheral blood of mice. Medium-dose and high-dose CLDENs significantly reversed the inhibitory effect of cyclophosphamide on blood cell counts (Fig. 6A-D). As for the low-dose group, CLDENs significantly alleviated the decline of white blood cells, lymphocytes, but no statistical difference was observed in granulocytes and monocytes (Fig. 6C and D).

Subsequently, to evaluate whether CLDENs still induced the secretion of inflammation-associated cytokines in vivo, the levels of the relevant cytokines in mouse serum were detected by ELISA assay. Very low levels of TNF-α were observed in the model group, and cyclophosphamide also inhibited the secretion of IL-2. Even in the presence of cyclophosphamide, CLDENs significantly increased serum TNF-α levels in vivo and exhibited a concentration dependence (Fig. 6E). Similarly, CLDENs significantly increased the level of IL-2 (Fig. 6F).

The CD4^+^/CD8^+^ ratio in mature T lymphocytes is considered to be a key indicator of the level of immunity in the body [61]. Mature T lymphocytes were isolated from the spleens of animals (CD3^+^). Cyclophosphamide significantly suppressed the number of CD3^+^CD4^+^ T cells (Fig. 6G) and decreased the ratio of CD4^+^/CD8^+^ T cells (Fig. 6H). Low-dose and medium-dose of CLDENs did not show a restorative effect on the absolute number of CD4^+^ T cells, but significantly increased the CD4^+^/CD8^+^ T cell ratio. In the high-dose group of CLDENs, both the number of CD4^+^ T cells and the ratio of CD4^+^/CD8^+^ T cells were significantly restored in the animals. These results suggested that CLDENs promoted positive regulation of immune function by improving the ratio of lymphocyte subpopulations.

Normal mouse Kupffer cells and spleen macrophages can remove foreign substances from the bloodstream. Blood was obtained at the 2nd and 10th minutes after the mice were given 25% Indian ink (a suspension of carbon particles) via tail vein, and the concentration of carbon particles in the blood was calculated. The function of macrophages in animals might be judged by calculating the clearance index *K* and phagocytosis coefficient α according to the speed at which carbon particles are cleared from the bloodstream. Experiments on carbon clearance revealed that cyclophosphamide dramatically decreased the clearance index *K* and phagocytosis coefficient α of mice (Fig. 6I and Table 1), which suggested that cyclophosphamide reduced the ability of mice to remove carbon particles from their blood circulation. After the administration of CLDENs (20 mg/kg and 60 mg/kg), the phagocytosis coefficient of cyclophosphamide immunosuppressed mice was dramatically increased, indicating that CLDENs had a positive protective effect on macrophage function. However, the animals in the low-dose group did not show a significant improvement in phagocytosis coefficient α.

**Table 1:** Effect of CLDENs on the carbon clearance experiment of cyclophosphamide-induced immunosuppression mice

Groups	Clearance index *K*	Phagocytosis coefficient *α*
Blank	0.0110 ± 0.0030	0.0868 ± 0.0227
Model	0.0019 ± 0.0005^*^	0.0127 ± 0.0032^*^
CLDENs-L	0.0054 ± 0.0022	0.0329 ± 0.0145^*^
CLDENs-M	0.0073 ± 0.0024^#^	0.0420 ± 0.0131^#^
CLDENs-H	0.0105 ± 0.0029^#^	0.0684 ± 0.0218^#^

Data were mean ± SD, n = 3; ^*^P < 0.05 *vs.* Blank; ^#^*P* < 0.05 *vs.* Model

The strong cell cycle arrest effect of cyclophosphamide is one of the inducements of its immunosuppression effect [17]. The distribution of the cell cycle was then examined using flow cytometry on bone marrow cells that had been extracted from mouse femurs. The findings demonstrated that CLDENs treatment alleviated the effect of cyclophosphamide-induced G1 arrest in mouse bone marrow cells (Fig. 6J).

It is reported that the transcription factor PU.1 determined HSC’s differentiation fate of macrophages, neutrophils, dendritic cells and lymphocytes [58]. The latest report found that inflammation-related cytokines such as IL-1 and TNF-α can rapidly activate HSC cell division and bone marrow differentiation through PU. 1 dependent gene program [62, 63]. In in vitro experiments, CLDENs showed immunostimulatory effects via TNF-α/ NF-κB/PU.1 axis. In the cyclophosphamide-induced immunosuppression mouse model, total proteins from mouse bone marrow cells were extracted for western blot assay. The outcomes demonstrated that CLDENs rescued the decrease of the expression level of PU. 1 protein induced by cyclophosphamide, but there was no significant increase compared with the blank group (Fig. 6K). When PU. 1 in relatively low levels, HSC are more likely to develop into lymphocytes, whereas when PU. 1 in relatively high levels, HSC are more likely to differentiate into myeloid cells [59]. This is in line with the rise in lymphocytes in peripheral blood and the weight of the spleen.

Animal body weight changes were tracked constantly throughout the administration period, starting with the initial body weight on the first day of administration. The body weight of the mice in the control group grew steadily over time. After being given cyclophosphamide, the animals' body weight was found to drop quickly (Fig. 6L, Day 9–11). The body weight of the animals in the model group remained unrecovered at the end of the dosing. In the CLDEN administration groups, this phenomenon gradually recovered as cyclophosphamide administration was discontinued. The CLDENs group had nearly reached its baseline body weight at all doses after 14 d of treatment.

Next, we investigated how cyclophosphamide affected animals’ immune organ (Table 2). Spleen and thymus weights were discovered to have significantly decreased after treated with cyclophosphamide. Although low doses of CLDENs did not restore the spleen weight to normal levels, the loss of spleen weight caused by cyclophosphamide in mice was dramatically reversed by the administration of CLDENs compared to the model group. The high dose of CLDENs even significantly increased the weight of the spleen compared to the blank control. However, Medium-dose and high-dose groups only attenuated cyclophosphamide-induced thymic weight loss to a limited extent. In the CLDENs low-dose group (6 mg/kg), an even more significant reduction in thymus weight was demonstrated, as well as a significant increase in liver weight.

**Table 2:** Effect of CLDENs on the organ index of cyclophosphamide-induced immunosuppression mice

Groups	Organ index (mg/g)				
	Spleen	Thymus	Heart	Liver	Kidney
Blank	2.38 ± 0.25	1.41 ± 0.82	5.45 ± 0.63	46.38 ± 8.38	16.13 ± 2.64
Model	1.42 ± 0.27^***^	0.67 ± 0.33^*^	5.46 ± 0.55	50.87 ± 2.81	16.13 ± 1.75
CLDENs-L	1.97 ± 0.22^*, ##^	0.46 ± 0.32^**^	5.27 ± 0.52	52.63 ± 4.00^*^	15.56 ± 0.95
CLDENs-M	2.50 ± 0.50^###^	0.82 ± 0.40	4.94 ± 0.79	51.62 ± 5.36	15.70 ± 1.71
CLDENs-H	3.39 ± 0.54^***, ###^	0.70 ± 0.48^*^	5.21 ± 0.78	50.06 ± 3.24	15.40 ± 1.41

Data were mean ± SD, n = 10; ^*^P < 0.05, ^**^P < 0.01 and ^***^P < 0.001 vs. Blank; ^##^*P* < 0.01 and ^###^*P* < 0.001 *vs.* Model

Hematoxylin–eosin staining was performed to detect histological changes in the liver and kidney. Liver and kidney damage was observed in the model group. Liver tissues of the model group showed lymphocyte substitution after liver cell necrosis (Fig. 6M, marked by black triangles), and the kidney tissues showed obvious glomerular atrophy and fibrosis (Fig. 6M, marked by white triangles). These lesions were not observed in the CLDENs administration group.

In conclusion, the above experimental data illustrated that CLDENs alleviated cyclophosphamide-induced immunosuppression. CLDENs significantly ameliorated cyclophosphamide-induced reduction in the number of immune cells, lower cytokine levels and decreased immune clearance. CLDENs had protective effects on cyclophosphamide-induced weight loss and liver and kidney injury. Moreover, CLDENs were found to strongly stimulate the secretion of TNF-α in vitro and in vivo. Although medium and high doses of CLDENs exhibited excellent immunomodulatory activity, we observed that co-administration of low-dose CLDENs and cyclophosphamide may carry some risk of organ toxicity. It has been reported that endocrine disruptors may have more complex dose–effect curves rather than linear monotonic dose responses [64]. We speculate that there may be a low-dose effect of CLDENs as a strong stimulator of TNF-α.

### Plant cell culture technology applied to CLDENs production

After determining the in vivo and in vitro immunomodulatory activity of CLDENs, we attempted to use plant cell culture techniques to achieve expanded production of CLDENs. First, *C. roseus* dedifferentiated cells and *C. roseus* cambial meristematic cells were induced from the leaves and tender stems of *C. roseus*, respectively (Additional file 1: Fig. S7A and B). Microscopically, *C. roseus* dedifferentiated cells had an irregular cell shape, forming in clusters and masses with a large intracellular vacuole (Additional file 1: Fig. S7C), whereas *C. roseus* cambial meristematic cells were spherical, distributed single cells with a very small vacuole (Additional file 1: Fig. S7D).

After expanded culture, the *C. roseus* cells were transferred to liquid medium. *C. roseus* cells were suspended and cultured in complete darkness for 1 week. Extracellular vesicles were obtained by ultracentrifugation and characterized within 48 h after extraction. We calculated the ratio of total vesicle protein amount to raw material mass. Nearly one gram of nanovesicles could be obtained from fifty liters of suspended cells. The yields of *C. roseus* dedifferentiated cells-derived exosome-like nanovesicles (CrDDENs) and *C. roseus* cambial meristematic cells-derived exosome-like nanovesicles (CrCDENs) were 2.58 ± 0.47 mg/g and 2.73 ± 0.84 mg/g, respectively, compared to CLDENs (0.80 ± 0.34 mg/g) (Additional file 1: Fig. S7E).

**Fig. 7:**
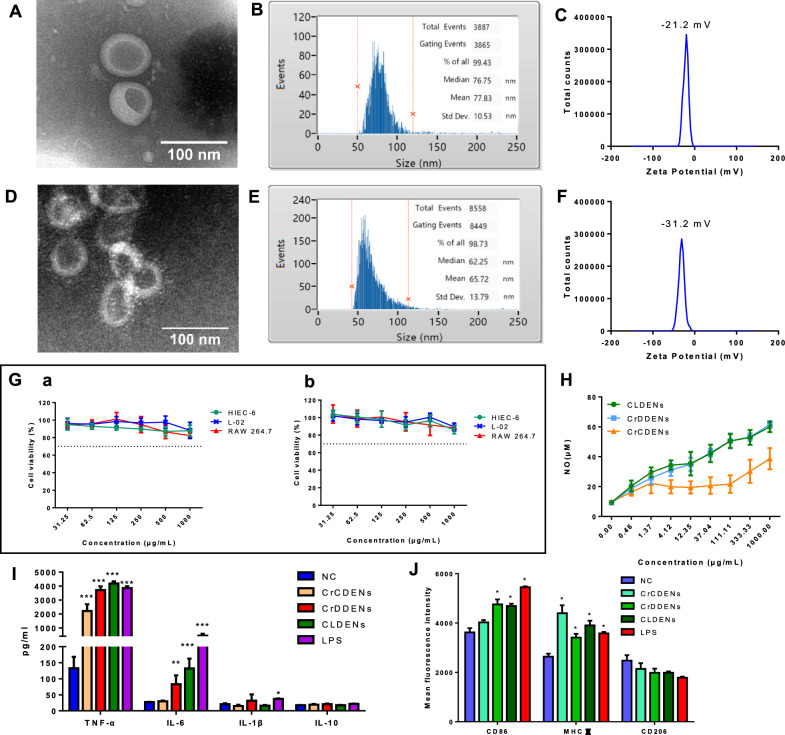
Plant cell culture technology applied to CLDENs production. **A** TEM analysis of CrDDENs. Scale bar = 100 nm. **B** Measurement of CrDDENs particle size distribution by nanoflow analyzer. **C** Zeta potential analysis of CrDDENs. **D** TEM analysis of CrCDENs. Scale bar = 100 nm. **E** Measurement of CrCDENs particle size distribution by nanoflow analyzer. **F** Zeta potential analysis of CrCDENs. **G** Cytotoxicity assay of CrDDENs and CrCDENs against multiple cell lines (n = 3). **H** CrDDENs and CrCDENs promoted nitric oxide release from RAW264.7 cells. **I** CrDDENs and CrCDENs promoted the secretion of cytokines from RAW264.7 cells. **J** CrDDENs induced the polarization of RAW264.7 cells toward M1 type. Data were mean ± SD, n = 3; ^*^P < 0.05, ^**^P < 0.01 and ^***^P < 0.001 *vs.* Control

The results showed that both CrDDENs and CrCDENs were circular vesicles (Fig. 7A and D), and the particle size of CrDDENs was 77.83 ± 10.53 nm (Fig. 7B) and that of CrCDENs was 65.72 ± 13.79 nm (Fig. 7E). The zeta potential of CrDDENs was −21.2 mV (Fig. 7C), and that of CrCDENs was −31.2 mV (Fig. 7F). It was found that CrDDENs had highly similar physical characteristics to CLDENs, while CrCDENs, from *C. roseus* stems, differed from CLDENs in terms of particle size and zeta potential.

Similar to CLDENs, both CrCDENs and CrDDENs exhibited low cytotoxic effects. After 48 h of treatment with CrCDENs and CrDDENs on HIEC-6 cells, L-02 cells, and RAW264.7 cells, the cell survival rates were all greater than 70% (Fig. 7G).

Subsequently, we evaluated the immunomodulatory activity of CrDDENs and CrCDENs. As shown in Fig. 7H, CLDENs, CrDDENs, and CrCDENs can significantly stimulate nitric oxide secretion from RAW264.7 cells in the range of 0–1000 μg/ml. Moreover, CrDDENs and CLDENs had similar concentration-effect curves. ELISA assays were used to examine the effects of CrDDENs and CrCDENs on the release of inflammatory cytokines from RAW264.7 cells. CrCDENs promoted the release of TNF-α from RAW264.7 cells, but had no significant effect on the secretion level of IL-6. Similar to CLDENs, CrDDENs strongly stimulated the release of TNF-α and IL-6 from RAW264.7 cells in vitro. The level of TNF-α in RAW cell culture medium supernatant increased from 132.86 ± 61.45 pg/ml to 3714.10 ± 452.2 pg/ml after administration of CrDDENs (Fig. 7I).

In addition, CrDDENs exhibited similar effects to CLDENs in promoting macrophage polarization. When 120 μg/ml CrDDENs was co-incubated with RAW264.7 cells for 24 h, the expression levels of CD86 and MHC II in RAW264.7 cells increased simultaneously, demonstrating that CrDDENs could also induce the polarization of RAW264.7 cells toward M1 type (Fig. 7J). In contrast, CrCDENs strongly enhanced the expression of MHC II, but had no significant effect on the expression of CD86.These data suggested that for different tissue sources of PDENs, their biological activities may not be identical.

Our results demonstrated that CLDENs-like nanovesicles can be isolated from dedifferentiated cells of *C. roseus*, and they have similar physical characteristics and biological activities. These data showed the feasibility of PDENs production by plant cell culture technology.

## Discussion

In this paper, we isolated a novel PDENs from *C. roseus* and studied their characteristics and biological activity. In contrast to earlier researches, we did not extract PDENs from the plant's juice but rather separated nanovesicles from different parts of the plant. In fact, our experimental results also demonstrated that nanovesicles from different parts of the plant performed differently. How to obtain relatively pure extracellular vesicles had been the primary problem faced by researchers. Utilize differential ultracentrifugation, density gradient centrifugation, magnetic bead sorting [65] and size exclusion chromatograph [21] have been used in extraction of PDENs. However, these methods still face the problems of inefficiency and incorrect separation of non-vesicular nanoparticles [13]. In this article, we proposed a method gently isolating extracellular vesicles from apoplastic fluid using cellulase and pectinase digestion without damaging plant cell structure. This approach of enzyme digestion can produce relatively pure extracellular vesicles. At the same time, in order to prepare CLDENs more efficiently, we tried to use plant cell culture technology.

Plant cell culture technology is widely used in plant breeding, reforestation, biosynthesis of natural products etc., and has formed mature experience in large-scale commercial production [66]. We induced dedifferentiated cells and cambial meristematic cells from the leaves and tender stems of *C. roseus*, and isolated extracellular vesicles from the liquid medium. By this method, we can easily obtain more than one gram of PDENs from plant cell culture medium supernatant. Besides, we found that a unified source of plant tissue is necessary for the production of PDENs. The similarity between CrDDENs and CLDENs were determined from both physical characteristics and biological activities, while CrCDENs were slightly different from CLDENs. Our research has proved the feasibility of applying plant cell culture technology to the production of PDENs, and it is worth looking forward to large-scale production of PDENs in the future.

Marker protein-based engineering of exosomes has been shown to be a drug delivery strategy with great potential [67]. Compared to mammalian exosomes, PDENs do not require a tightly controlled culture environment and clearly have lower production costs. However, due to the diversity of plants and the complexity of their own tissues, the research on the composition analysis of and their biogenetic mechanism is still in their infancy. For the first time, differential proteomics were used to analyze the difference between CLDENs and their source plant tissues, and six marker proteins of CLDENs were identified. Customized modification strategy based on marker proteins will become an important direction for future applications of CLDENs. Additionally, high enrichment of MVB-related proteins identified in differential proteomics supported the possibility that CLDENs were derived from plant cell MVB. The experimental results will hopefully serve as useful feedback information for the biogenesis mechanism of PDENs.

In this paper, the special lipid composition of CLDENs were reported, which had an unusually high content of ether-phospholipids (more than 30%). As mentioned earlier, ether phospholipids provide better rigidity for exosomes and may be involved in inflammatory signaling processes [45]. It is also reported that granulocytes and monocytes have higher levels of ether-phospholipids compared to other blood cells [68], which may point to a link between lipid composition and immune cell function. One of the identified ether phospholipids, platelet activating factor (PAF), is a powerful mediator of inflammation [69]. Our result ties well with previous studies that CLDENs did have unique immune-enhancing effects in vivo and in vitro, and demonstrated excellent stability in various cruel environments. Besides, our research also indicated that the structural integrity of CLDENs is essential for its immunomodulatory activity (Additional file 1: Fig. S6). The immunomodulatory function of exosome lipids is an interesting area that has not yet been explored. Unfortunately, the ether-phospholipid component isolated from CLDENs has not yet reached quantities sufficient to support validation activity experiments. Despite its preliminary character, this study indicated that lipid composition is a noteworthy issue when studying the bioactive effects of mammalian exosomes or PDENs, especially immune-related biological processes.

Pharmacological activity is the key to PDENs applications. According to our data, CLDENs strongly stimulated the secretion of TNF-α in vivo and in vitro*,* while CrDDENs and CrCDENs showed similar phenomena. As an inflammatory mediator, TNF-α has been shown to promote the conversion of myeloid leukemia cells to macrophages and has been used in combination with all-trans retinoic acid to treat myeloid leukemia [70, 71]. It is reported that TNF-α can directly promote PU.1 up-regulation, myelopoiesis, and prevents necroptosis of HSC [31]. These findings demonstrate the potential of inflammatory factors to regulate hematopoietic differentiation processes. Giving appropriate inflammatory signals to stimulate HSC differentiation may be a viable strategy to cope with acute myelosuppression during chemotherapy. Moreover, CLDENs enhanced the phagocytic activity of macrophages, promoted proliferation of lymphocytes, and up-regulated the expression of the hematopoietic function-related transcription factor PU.1 both in vivo and in vitro. Based on the targeted distribution of CLDENs in immune organs after intraperitoneal injection, we used intraperitoneal injection in animal experiments. We hypothesized that CLDENs could be taken up by immune organs and HSC, and exert proliferative and functional activation effects on immune cells. In fact, CLDENs did alleviate multiple indications of cyclophosphamide-induced immunosuppression, showing a restorative effect on post-chemotherapy myelosuppression. In sum, CLDENs were promising as a potential chemotherapeutic immune cofactor for improving hematopoietic function and enhancing immune response.

## Conclusions

In this paper, novel PDENs were isolated from *Catharanthus roseus* leaves (CLDENs). A high content of ether-phospholipids, MVB-related proteins, and six marker proteins were identified in CLDENs. These findings provided inspiration for the biological formation and activity tracking of PDENs. CLDENs could be internalized by macrophages, and stood to severe pH environments and various enzyme digestions. Furthermore, CLDENs had immune organ targeting after intraperitoneal injection and alleviated cyclophosphamide-induced immunosuppression via TNF-α/ NF- κ B/PU.1 axis. To ensure a steady supply of CLDENs, plant cell culture systems of *C. roseus* were established to provide CLDENs-like nanovesicles which had similar physical properties and biological activities. Our research indicated that CLDENs might be a potential immunomodulatory material, and can be produced at scale for industrial application.

## Materials and methods

### Cell lines

The cell lines needed for the experiment were purchased from the National Collection of Authenticated Cell Cultures (Shanghai, CHN). Cells were cultured in Dulbecco’s Modified Eagle’s Medium (DMEM) or RPMI 1640, supplemented with 10% foetal bovine serum, 10% antibiotics (100 U/ml penicillin and 100 mg/ml streptomycin). All cells were incubated at 37 °C in a humidified atmosphere with 5% CO_2_.

### Isolation of the exosome-like nanovesicles

For CLDENs, fresh *C. roseus* leaves were washed to remove sludge and weighed for recording. The leaves were digested with cellulase (3 g/100 ml) and pectinase (0.2 g/100 ml) for 12 h, the mixture was centrifuged at 3000 rpm for 20 min to remove the protoplasts. Then, the supernatant was centrifuged to remove plant fibers (1000 rpm, 40 min, 4 °C) and concentrated to about 100 ml by hollow fiber module.

*C. roseus* dedifferentiated cells and *C. roseus* cambial meristematic cells were induced using the methods reported previously [72, 73]. Two gram of well-grown *C. roseus* cells were placed in 100 ml of liquid medium (containing 4.43 g/l Murashige and Skoog culture medium, 20 g/l sucrose and 2 mg/l 1-naphthlcetic acid). Five liters of *C. roseus* dedifferentiated cells and *C. roseus* cambial meristematic cells were incubated in suspension for 7 days in complete darkness with slow shaking at 120 rpm. Then, the *C. roseus* cells were filtered through gauze, and the medium was collected. Impurities were removed by centrifuge at 10,000 g for 10 min at 4 °C, and the supernatant was collected. Subsequently, the supernatant was concentrated using hollow fiber module.

The sample that had been concentrated by the hollow fiber module was placed in the ultra-clean ultracentrifuge tube after 7 ml of 0.971 M sucrose was added to the bottom. The sucrose layer was collected and diluted by sterile phosphate buffered saline (PBS) after centrifuged at 150,000 × g for 90 min at 4 °C. Excess sucrose was removed using an ultrafiltration tube. Finally, the nanovesicles were resuspended in PBS.

The newly extracted nanovesicles were quantified based on protein concentration using a BCA protein quantitative kit (Beyotime, Shanghai, CHN). For long-term storage, they will be stored at −80 °C until use.

### Physicochemical characterization

The exosome-like nanovesicles were absorbed onto a carbon-coated copper grid and dyed for 90 s with 3% phosphotungstic acid, detected by transmission electron microscope (JEM-1400Flash, JEOL, Tokyo, Japan). The size distribution was analyzed using Nanoflow detector (NanoFCM, Xiamen, CHN). The DLS detection and zeta potential of nanovesicles were assessed using Zetasizer Nano ZS (Malvern Instruments Ltd., Malvern, UK).

For the stability evaluation procedure, CLDENs were prepared of the same batch and digested with DNase (3 μg/ml), RNase (6 μg/ml) and proteinase K (100 μg/ml) in 37 °C for 30 min. CLDENs were incubated with 0.02% Triton X-100 and 0.1% SDS solution for 30 min at room temperature to digest the lipids in the samples. CLDENs were incubated with NaOH(pH > 13) and HCl (pH < 2) at room temperature for 30 min. A portion of the processed CLDENs were directly subjected to particle size distribution measurements. The rest samples were treated with enzyme inactivation, surfactant blocking and acid–base neutralization treatment to examine the effect of CLDENs on the ability of RAW264.7 cells to secrete nitric oxide.

The small intestine simulating fluid contained 6.8 g/l KH_2_PO_4_, 10 g/l pancreatic enzyme, and was adjusted the pH to 6.8 using 0.2 M NaOH and 0.2 M HCl. The stomach simulating fluid contained 2.0 g/l NaCl, 3.2 g/l pepsin (800–2500 IU/mg) and was adjusted pH to 2.0 using HCl. CLDENs were submitted to DLS analysis after being cultured for a predetermined amount of time in small intestine simulating fluid and stomach simulating fluid at 37 °C.

### Laser scanning confocal microscopy analysis

CLDENs were incubated with PKH26 probe for 30 min at 37 °C. CLDENs-PKH26 were obtained by exosome extraction kit (Bestbio, Shanghai, CHN). RAW264.7 cells were treated with 1 g/ml CLDENs-PKH26 for 4, 24 and 48 h, respectively. Laser scanning confocal microscopy (LSM800, Zeiss, Oberkochen, GER) was used to observe the uptake of exosome-like nanoparticles by cells.

### Cytotoxicity assay

Cytotoxicity of CLDENs in vitro was measured with MTT assay according to the references [74]. CLDENs (31.25–1000 µg/ml) were incubated with cells for 48 h, then cell viability was calculated.

Mouse spleen lymphocytes were extracted according to the instructions of the Mouse Spleen Lymphocyte Isolation Solution Kit (Solarbio, Beijing, CHN). Cells were inoculated in 96-well plates at 5.0 × 10^6^ cells/well and incubated with CLDENs (7.81–1000 µg/ml) for 48 h. Then, cell viability was assayed by Cell Counting Kit-8 (Yeasen, Shanghai, CHN).

### Hemo-compatibility assay

Hemo-compatibility assay was performed according to the references [75, 76]. Briefly, sheep blood was mixed with 0.05,0.1,0.5,1,2,4,8 mg/mL of CLDENs at 1:1 volume and incubated at 37 °C for 30 min, the OD 425 nm of the supernatant was then detected.

### Determination of nitric oxide

The cells were plated into a 96-well plate at 1 × 10^5^ cells/well for 24 h and exposed to CLDENs, CrDDENs or CrCDENs for 24 h at 37 °C and 5% CO_2_. The culture medium and LPS (1 μg/ml) were used as blank and positive controls, respectively. Total nitric oxide assay kit (Beyotime, Shanghai, CHN) was used to detect the content of nitric oxide in the culture supernatant. The values were calculated according to the calibration curve with NaNO_2_.

### Flow cytometry

The detection of macrophage surface antigen molecule was used flow cytometry. RAW264.7 cells were plated into a 12-well plate at 2 × 10^4^ cells/well for 24 h, and then were treated with CLDENs, CrDDENs, CrCDENs or LPS. After 24 h of incubation, cells were suspended and incubated with FITC-anti-CD86 antibody (Biolegend, CA, USA), APC-anti-CD206 antibody (Biolegend, CA, USA), PE-anti-MHC II antibody (Invitrogen, Carlsbad, USA) at room temperature for 15 min, and then tested by flow cytometer.

Detection of FITC-dextran internalization was performed to investigate the phagocytosis of RAW264.7 cells. RAW264.7 cells exposed to different concentrations of CLDENs were collected. After incubated with FITC-dextran (1 mg/ml, Angfeibio, Guangzhou, CHN) at 37 °C for 1 h, RAW264.7 cells were treated with cold PBS to terminate the reaction. Then, cells were washed and re-suspended in PBS. The uptake of FITC-dextran in RAW264.7 cells was detected by flow cytometry.

### Measurement of cytokine production

RAW264.7 cells were plated into a 96-well plate at 1 × 10^6^ cells/well, and then were treated with CLDENs, CrDDENs, CrCDENs or LPS 24 h. After another 24 h of incubation, the cell culture supernatant was collected and the concentration of TNF-α, IL-6, IL-1β, IL-10 were assessed using ELISA kits (ExCell, Shanghai, CHN).

Mouse spleen lymphocytes were extracted according to the instructions of the Mouse Spleen Lymphocyte Isolation Solution Kit (Solarbio, Beijing, CHN). Lymphocytes were inoculated in 96-well plates at 5.0 × 10^6^ cells/well and incubated with CLDENs (30–240 µg/ml) for 24 h. Then, the cell culture supernatant was collected and the concentration of IL-2 were assessed using ELISA kits (ExCell, Shanghai, CHN).

### Real-time quantitative PCR analysis

Real-time quantitative PCR was performed with reference to the literature [77]. All reactions were normalized to GAPDH levels. Primer sequences were shown in Additional file 1: Table S5.

### Immunoblotting

The samples were analyzed using the WES system (ProteinSimple, San Jose, CA, USA) and western blot assay [78]. Anti-NF-κB antibody, anti-phospho-NF-κB (Ser536) antibody, anti-PU.1/Spi1 antibody were purchased from Affinity Biosciences (Cincinnati, USA). Anti-iNOS antibody, anti-β-actin antibody and anti-GAPDH antibody were purchased from Cell Signaling Technology (Danvers, USA).

### Animal experiment

BALB/c mice (4–5 weeks of age, male) were purchased from Guangdong GemPharmatech Co., Ltd (Guangdong, China) with the quality certificate number of 44824700013424. Before formal experimentation, each animal was housed in a metabolic cage for 10 days to allow for acclimation to the environment. Throughout the experiments, all the animals had free access to water and food and were housed in an environmentally controlled breeding room (temperature of 20 ~ 25 °C, relative humidity of 50 ~ 60%) with a 12 h light–dark cycle.

For the biodistribution assay of sample, CLDENs were incubated with DIR (Yeasen, Shanghai, China) at 37 °C for 30 min according to the instructions provided by the trial manufacturer. CLDENs-DIR were precipitated using an exosome extraction kit (Bestbio) and resuspended in PBS. Twenty-one BALB/c mice were randomly divided into oral administration group, intraperitoneal injection group and intravenous injection group. Mice were given a single dose of 60 mg/kg CLDENs-DIR by gavage, intraperitoneal injection, and tail vein injection, respectively. Subsequently, the distribution of CLDENs-DIR in vivo was detected by an animal imaging system in vivo (PerkinElmer IVIS Lumina Series III, PerkinElmer). The mice were dissected at the indicated time, and the heart, kidney, lung, brain, thymus, spleen, liver, and gastrointestinal tract were taken for fluorescence detection after washed by saline.

To establish cyclophosphamide-induced immunosuppression mice model, fifty BALB/c mice were randomly divided into blank group, model group, CLDENs-6 mg/kg group, CLDENs-20 mg/kg group and CLDENs-60 mg/kg group. Mice were given intraperitoneal injections of physiological saline (0.1 ml/10 g, once daily) for a total of 14 days in the blank control and model groups. For the CLDENs groups, mice received CLDENs intraperitoneally once daily for 14 days at doses of 6, 20 and 60 mg/kg body weight, respectively. Additional intraperitoneal injections of cyclophosphamide (80 mg/kg, 0.1 ml/10 g body weight) were administered on days 8, 9 and 10 of the treatment to the model group and the CLDENs groups once daily. Additional physiological saline (0.1 ml/10 g body weight) was intraperitoneally administered once day into the blank group. Two intraperitoneal injections should be spaced apart by at least 4 h on the same day.

Five mice from each group were randomly selected for the carbon clearance experiment [79]. Then, phagocytosis coefficient *α* and clearance index *K* were calculated. For the remaining mice, blood was collected for biochemical analysis. The expression of serum cytokines of mice was detected by ELISA kits. Bone marrow cells were collected for cell cycle assay and western blot experiment. After isolation of splenic lymphocytes from animal spleens, CD3^+^CD4^+^/CD3^+^CD8^+^ subpopulation ratio analysis was performed by flow cytometry. The liver and kidney were fixed in 4% formalin, embedded in paraffin, and sections were stained with hematoxylin and eosin (H&E). For all the dissected mice in each group, the thymus, spleen, liver, heart and kidney were taken and the organ index was calculated.

### Proteomic, lipidomic, metabolomic analysis and bioinformatics analysis

CLDENs (500 μl) was stored at −80 °C before lipidomic and metabolomic analysis by Beijing Bio-Tech Pack Technology Company Ltd. Disease correlation analysis of the identified compounds was performed based on the BATMAN-TCM online analysis tool (http://bionet.ncpsb.org.cn/batman-tcm/, #Job:batman-I2023-01-19-13886-1674095471). Proteomic analysis was application by Hangzhou Jingjie Biotechnology Co., Ltd.

### Statistical analysis

Results were expressed as mean ± standard deviation (SD), n ≥ 3. Two-tailed Student t-tests were used to assess differences between two groups, and two-way analyses of variance (ANOVA) were used to count differences between multiple groups. *P* < 0.05 was considered as a level of statistically significant difference.

## Supplementary Information

**Additional file 1: Figure S1.** Isolation of exosome-like extracellular vesicles from *Catharanthus roseus*.*C. roseus* picked from Guangzhou, China.TEM analysis of *C. roseus-*derived exosome-like nanovesicles. Scale bar = 100 nm.Particle size distribution of *C. roseus-*derived exosome-like nanovesicles. Peak 1: 200.80 ± 87.22 nm, with an intensity of 65.0%; Peak 2: 14.65 ± 3.40 nm, with an intensity of 34.0%; Peak 3: 4881.00 ± 682.00 nm, with an intensity of 1.0%.TEM analysis of exosome-like nanovesicles derived from *C. roseus* leaves, stems, and flowers. Scale bar = 100 nm.Protoplasts that settle at the bottom of the beaker after enzymatic digestion.Pictures of nanovesicles obtained by different ultracentrifugation methods. a. Juicing then differential ultracentrifugation; b. enzyme digestion then differential ultracentrifugation; c. juicing then sucrose cushion ultracentrifugation; d. enzyme digestion then sucrose cushion ultracentrifugation. **Figure S2**. CLDENs with membrane fusion in an acidic environment.The larger vesicle was fusing with the smaller vesicles.Two vesicles with similar particle sizes were merging. **Figure S3.** Biodistribution of CLDENs.Biodistribution of CLDENs in the organs after oral administration. a. Fluorescent signals in the brain, heart, liver, spleen, thymus, lung and kidney. b. Fluorescent signals in the gastrointestinal tract. A strong fluorescence signal exceeding the detection threshold of the instrument was observed in the stomach until the 12th hour.Biodistribution of CLDENs in the organs after tail vein injection. a. Fluorescent signals in the brain, heart, liver, spleen, thymus, lung and kidney. b. Fluorescent signals in the gastrointestinal tract. **Figure S4.** CLDENs were found in the lymph nodes in the neck of animals after intraperitoneal injection. **Figure S5.** Molecular weights distribution of the identified proteins in the PLANT group. **Figure S6.** Effects of different treatments on CLDENs’ immunostimulatory activity. CLDENs were treated with proteinase K at 37 °C, RNase A at 37 °C, DNase I at 37 °C, strong acid and strong base at 25 °C, 0.1% Triton X-100 at 25 °C, and 0.1% SDS at 25 °Cfor 30 min or heated at 100 °C for 10 min. Next, CLDENs’ ability to encourage nitric oxide secretion from RAW264.7 cells was investigated. CLDENs’ activity was not significantly altered by proteinase K at 37 °C, RNase at 37 °C, strong acid/strong base at 25 °C, and DNase I at 37 °C treatment, but their immunostimulatory activity was lost by 0.1% Triton X-100 at 25 °C, 0.1% SDS at 25 °C, and 100 °C heating treatment. Data were mean ± SD, n = 3; ^**^P < 0.01 and ^***^P < 0.001 *vs.* Blank. ^##^*P* < 0.01 and ^###^*P* < 0.001 *vs.* CLDENs group. ns, not significant. **Figure S7.** Induction of *C. roseus* cells.Germinating dedifferentiated cells in the leaves of *C. roseus*.Germinating cambial meristematic cells in the stems of *C. roseus*.*C. roseus* dedifferentiated cells viewed through a 20 × optical microscope. Scale bar = 100 μm.*C. roseus* cambial meristematic cells viewed through a 20 × optical microscope. Scale bar = 100 μm.. Comparison of the yield of three nanovesicles. Data were mean ± SD, n = 3, ^**^P < 0.01 *vs.* CLDENs group. **Table S1.** List of identified substances in lipidomic analysis. **Table S2.** List of identified substances in metabolomic analysis. **Table S3.** List of disease phenotypes that correlated with CLDENs. **Table S4.** Proteins significantly up-regulated in differential proteomics. **Table S5. **Real-time quantitative PCR primer sequence.

## Data Availability

The datasets used and/or analyzed during the current study are available from the corresponding author on reasonable request.

## Abbreviations

CLDENs: *Catharan**thus** roseus* (L.) Don leaves-derived exosome-like nanovesicles
CrCDENs: *C. roseus* cambial meristematic cells-derived exosome-like nanovesicles
CrDDENs: *C. roseus* dedifferentiated cells-derived exosome-like nanovesicles
CSF: Colony-stimulating factor
DLS: Dynamic light scattering
HSC: Hematopoietic stem cells
IL-1β: Interleukin-1β
IL-2: Interleukin-2
IL-6: Interleukin-6
IL-10: Interleukin-10
PBS: Phosphate buffered saline
PDENs: Plant-derived exosomes-like nanovesicles
TEM: Transmission electron microscopy
