# Supplementary material for: Novel plant-derived exosome-like nanovesicles from Catharanthus roseus: preparation, characterization, and immunostimulatory effect via TNF-α/NF-κB/PU.1 axis
Source: J Nanobiotechnology. 2023 May 20;21:160. doi: 10.1186/s12951-023-01919-x (PMC10199296; doi:10.1186/s12951-023-01919-x)
Supplement: Supplementary file 1 — Additional file 1: Figure S1. Isolation of exosome-like extracellular vesicles from Catharanthus roseus.C. roseus picked from Guangzhou, China.TEM analysis of C. roseus-derived exosome-like nanovesicles. Scale bar = 100 nm.Particle size distribution of C. roseus-derived exosome-like nanovesicles. Peak 1: 200.80 ± 87.22 nm, with an intensity of 65.0%; Peak 2: 14.65 ± 3.40 nm, with an intensity of 34.0%; Peak 3: 4881.00 ± 682.00 nm, with an intensity of 1.0%.TEM analysis of exosome-like nanovesicles derived from C. roseus leaves, stems, and flowers. Scale bar = 100 nm.Protoplasts that settle at the bottom of the beaker after enzymatic digestion.Pictures of nanovesicles obtained by different ultracentrifugation methods. a. Juicing then differential ultracentrifugation; b. enzyme digestion then differential ultracentrifugation; c. juicing then sucrose cushion ultracentrifugation; d. enzyme digestion then sucrose cushion ultracentrifugation. Figure S2. CLDENs with membrane fusion in an acidic environment.The larger vesicle was fusing with the smaller vesicles.Two vesicles with similar particle sizes were merging. Figure S3. Biodistribution of CLDENs.Biodistribution of CLDENs in the organs after oral administration. a. Fluorescent signals in the brain, heart, liver, spleen, thymus, lung and kidney. b. Fluorescent signals in the gastrointestinal tract. A strong fluorescence signal exceeding the detection threshold of the instrument was observed in the stomach until the 12th hour.Biodistribution of CLDENs in the organs after tail vein injection. a. Fluorescent signals in the brain, heart, liver, spleen, thymus, lung and kidney. b. Fluorescent signals in the gastrointestinal tract. Figure S4. CLDENs were found in the lymph nodes in the neck of animals after intraperitoneal injection. Figure S5. Molecular weights distribution of the identified proteins in the PLANT group. Figure S6. Effects of different treatments on CLDENs’ immunostimulatory activity. CLDENs were treated wit [file 12951_2023_1919_MOESM1_ESM.docx]

**Supplementary materials**

**Novel plant-derived exosome-like nanovesicles from *Catharanthus roseus*: Preparation, characterization, and** **immunostimulatory effect *via* TNF-α/NF-κB/PU.1 axis**

**Xiaozheng Ou^a,b,1^, Haoran Wang^c,1^, Huilin Tie^a^, Jiapei Liao^a^, Yuanyuan Luo^b^, Weijuan Huang^b^, Rongmin Yu^a,c,*^, Liyan Song^b,*^, Jianhua Zhu^a,*^**

*^a^ Biotechnological Institute of Chinese Materia Medica, Jinan University, Guangzhou 511443, China*

*^b^ Department of Pharmacology, Jinan University, Guangzhou 511443, China.*

*^c^ Weihai Neoland Biosciences Co., Ltd, Weihai 264209, China.*

^1^ *These authors contributed equally to this work.*

**^*^**Corresponding authors. Tel: +86-20-85220386, Fax: +86-20-85224766.

E-mail address: tyrm@jnu.edu.cn (Rongmin Yu), tsly@jnu.edu.cn (Liyan Song), tzhujh@jnu.edu.cn (Jianhua Zhu).


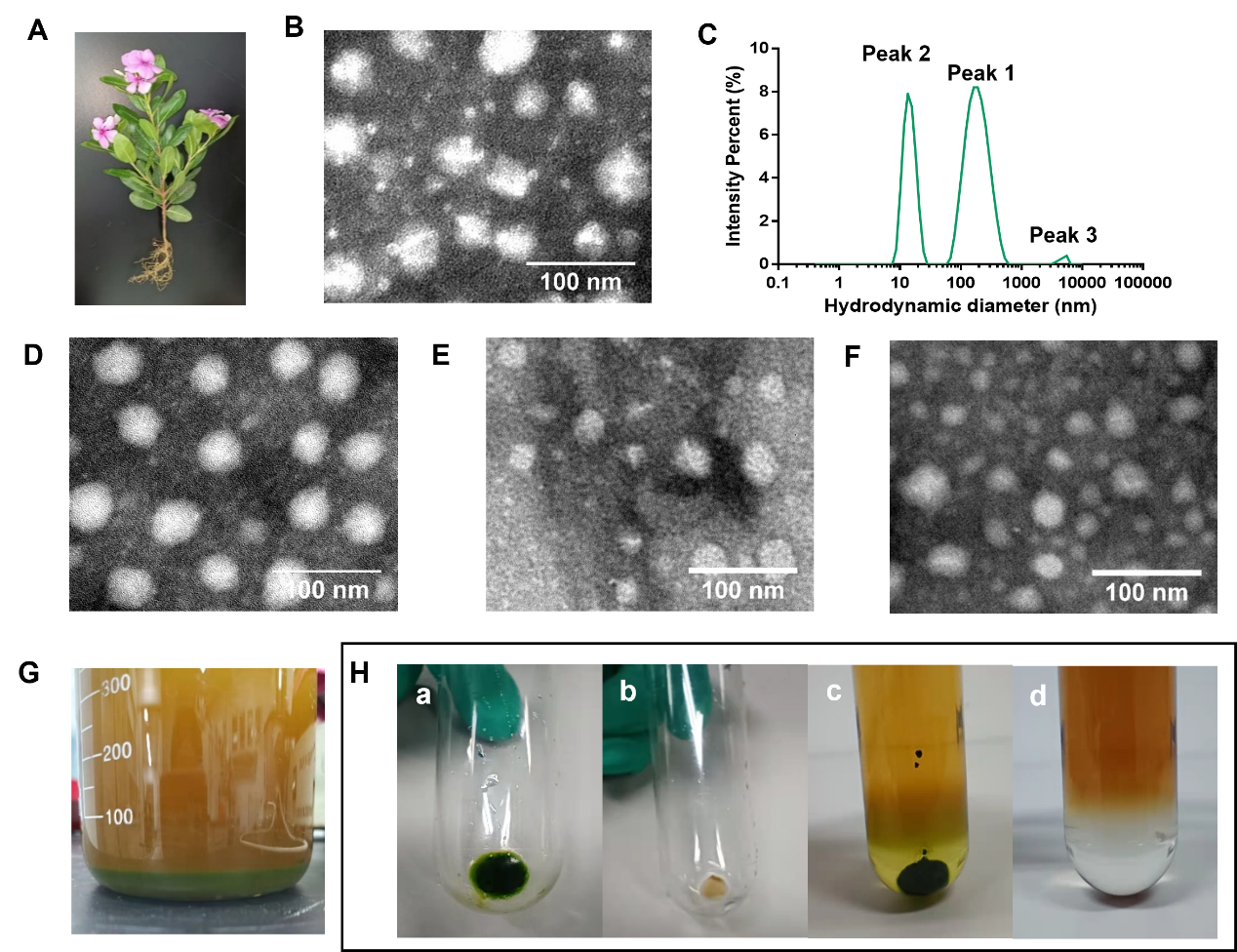


**Figure S1** Isolation of exosome-like extracellular vesicles from *Catharanthus roseus*. (A) *C. roseus* picked from Guangzhou, China. (B) TEM analysis of *C. roseus-*derived exosome-like nanovesicles. Scale bar = 100 nm. (C) Particle size distribution of *C. roseus-*derived exosome-like nanovesicles. Peak 1: 200.80 ± 87.22 nm, with an intensity of 65.0%; Peak 2: 14.65 ± 3.40 nm, with an intensity of 34.0%; Peak 3: 4881.00 ± 682.00 nm, with an intensity of 1.0% (n=3). (D-F) TEM analysis of exosome-like nanovesicles derived from *C. roseus* leaves (D), stems (E), and flowers (F). Scale bar = 100 nm. (G) Protoplasts that settle at the bottom of the beaker after enzymatic digestion. (H) Pictures of nanovesicles obtained by different ultracentrifugation methods. a. Juicing then differential ultracentrifugation; b. enzyme digestion then differential ultracentrifugation; c. juicing then sucrose cushion ultracentrifugation; d. enzyme digestion then sucrose cushion ultracentrifugation.


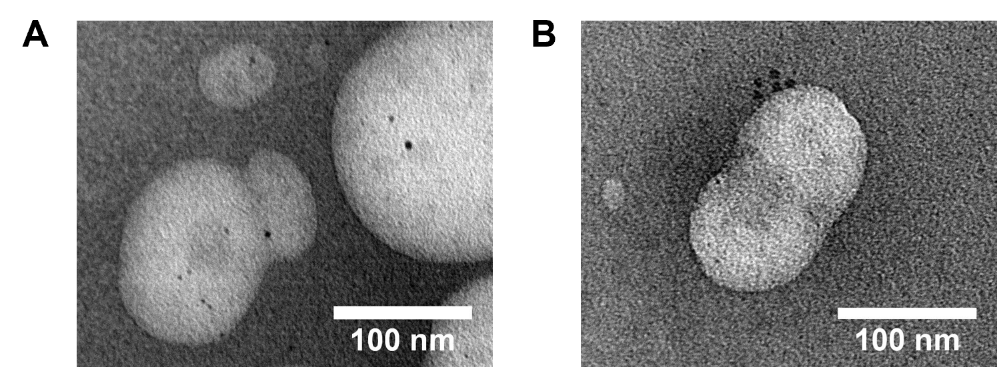


**Figure S2** CLDENs with membrane fusion in an acidic environment. (A) The larger vesicle was fusing with the smaller vesicles. (B) Two vesicles with similar particle sizes were merging.


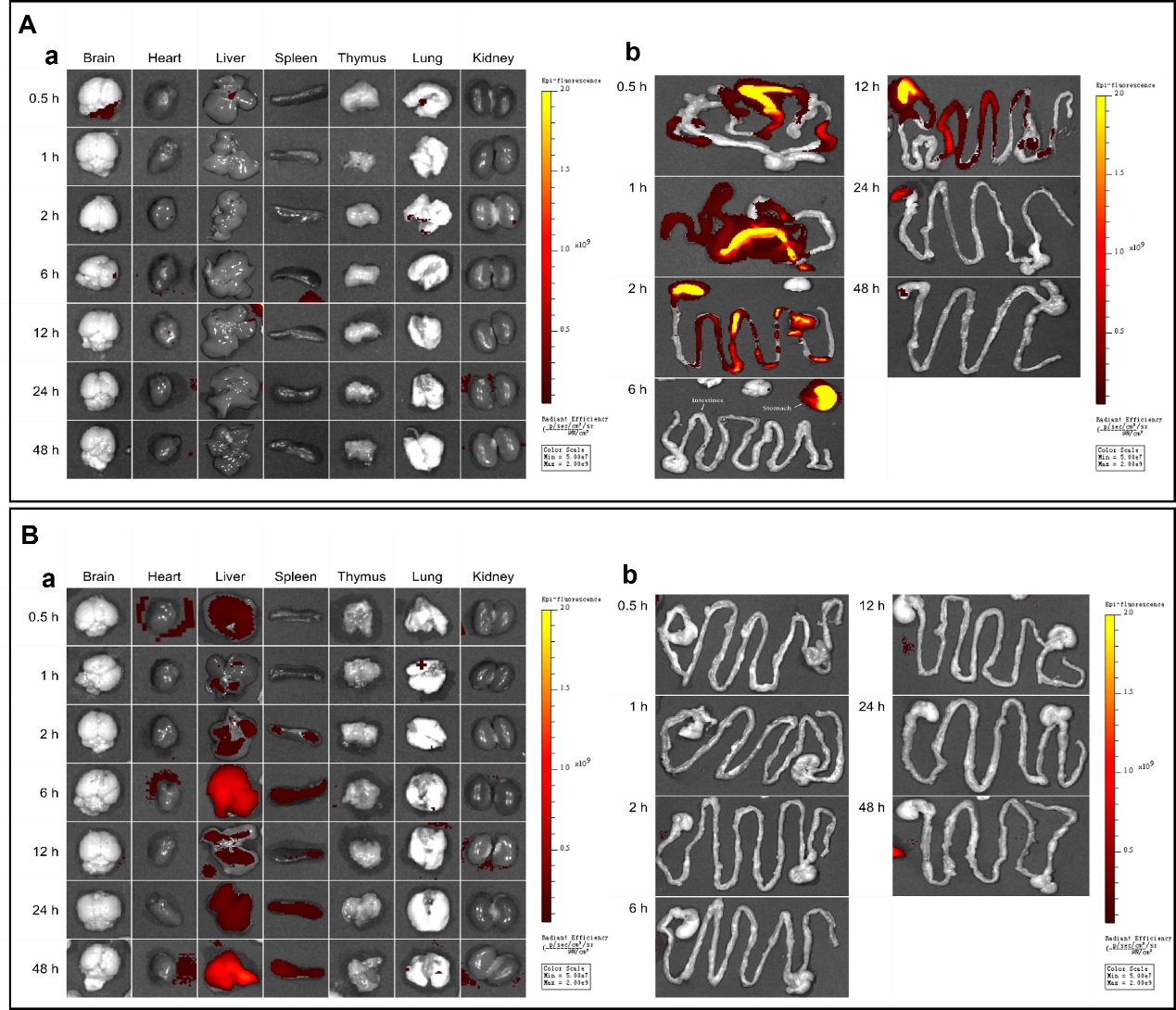


**Figure S3** Biodistribution of CLDENs. (A) Biodistribution of CLDENs in the organs after oral administration. a. Fluorescent signals in the brain, heart, liver, spleen, thymus, lung and kidney. b. Fluorescent signals in the gastrointestinal tract. A strong fluorescence signal exceeding the detection threshold of the instrument was observed in the stomach until the 12th hour (the fluorescence signal was greater than 3 ×10^10^). (B) Biodistribution of CLDENs in the organs after tail vein injection. a. Fluorescent signals in the brain, heart, liver, spleen, thymus, lung and kidney. b. Fluorescent signals in the gastrointestinal tract.

**Figure S4** CLDENs were found in the lymph nodes in the neck of animals after intraperitoneal injection.


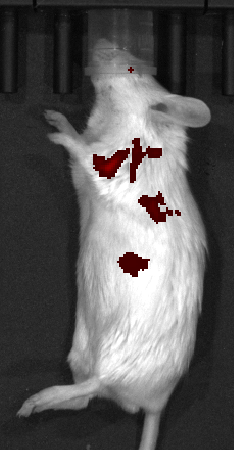

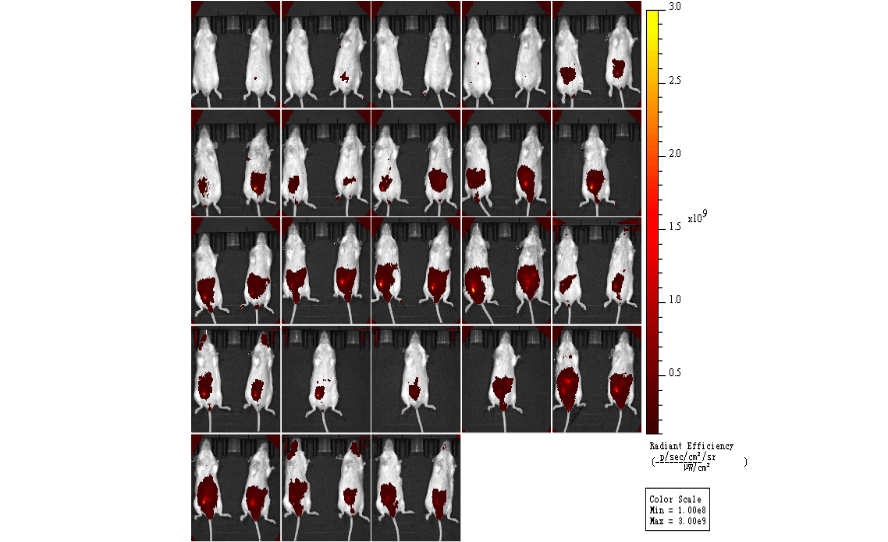


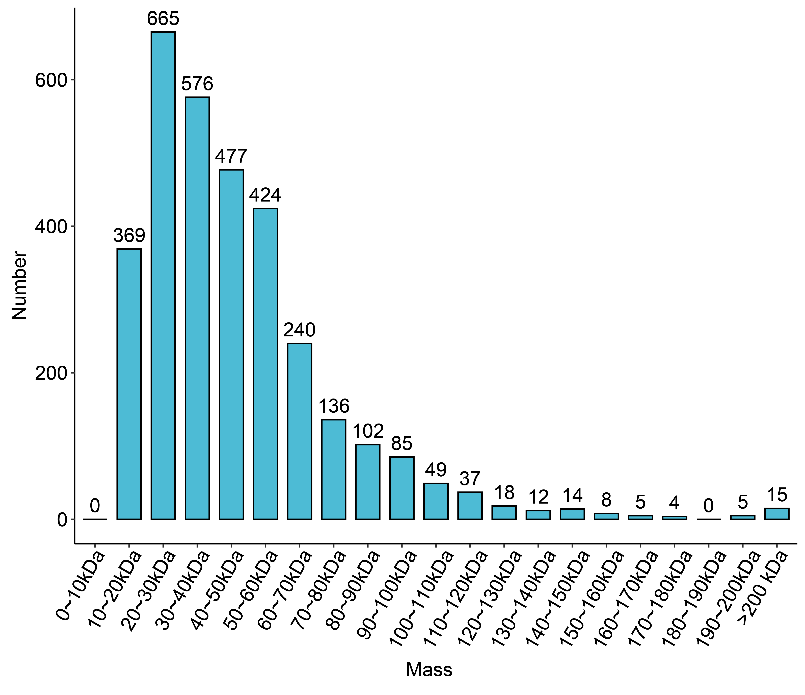


**Figure S5** Molecular weights distribution of the identified proteins in the PLANT group.


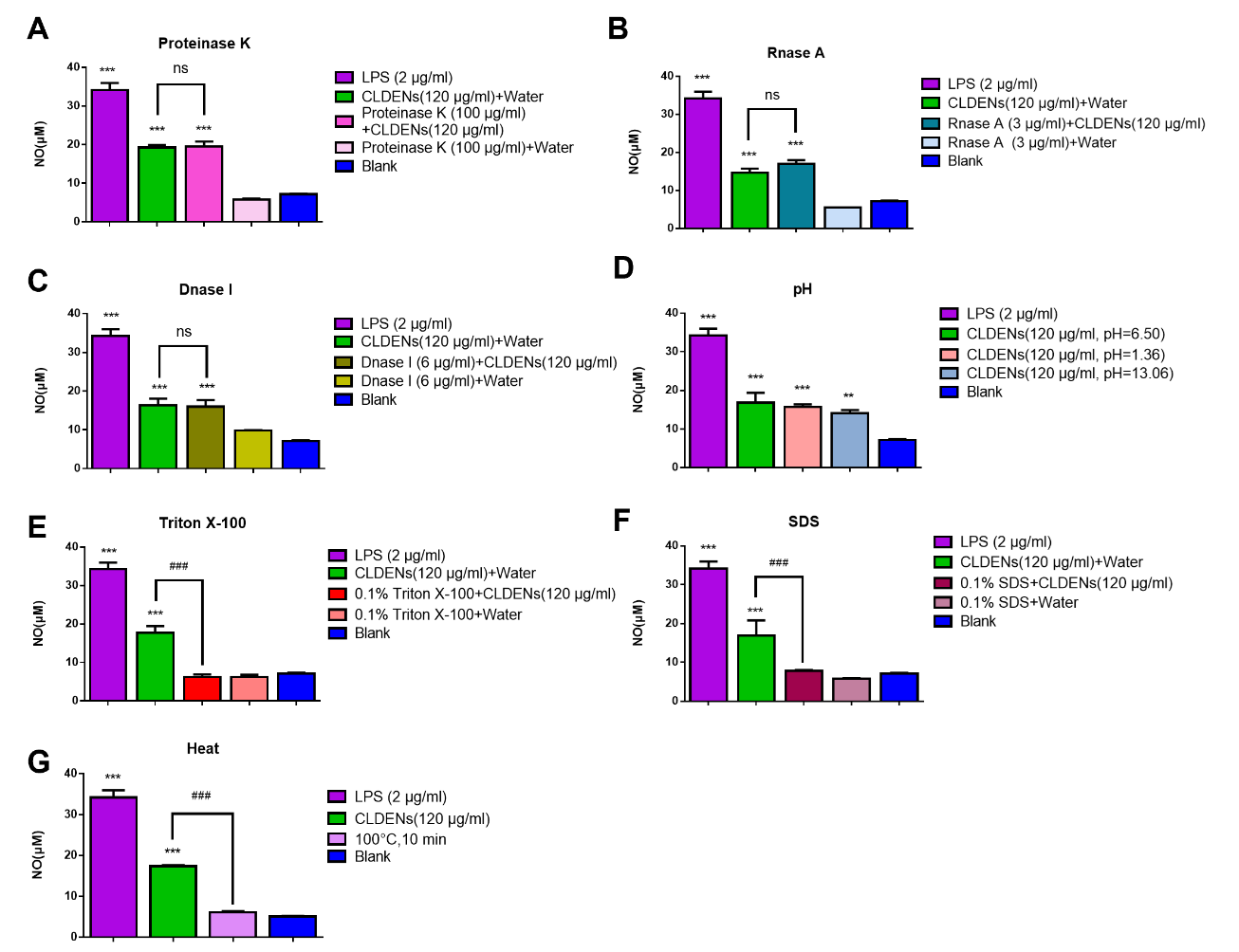


**Figure S6** Effects of different treatments on CLDENs’ immunostimulatory activity. CLDENs were treated with proteinase K at 37°C (A), RNase A at 37°C (B), DNase I at 37°C (C), strong acid and strong base at 25°C (D), 0.1% Triton X-100 at 25°C (E), and 0.1% SDS at 25°C (F) for 30 min or heated at 100°C for 10 min (G). Next, CLDENs’ ability to encourage nitric oxide secretion from RAW264.7 cells was investigated. CLDENs’ activity was not significantly altered by proteinase K at 37°C, RNase at 37°C, strong acid/strong base at 25°C, and DNase I at 37°C treatment, but their immunostimulatory activity was lost by 0.1% Triton X-100 at 25°C, 0.1% SDS at 25°C, and 100°C heating treatment. Data were mean ±SD, n=3; ^**^*P*<0.01 and ^***^*P*<0.001 *vs.* Blank. ^##^*P*<0.01 and ^###^*P*<0.001 *vs.* CLDENs group. ns, not significant.


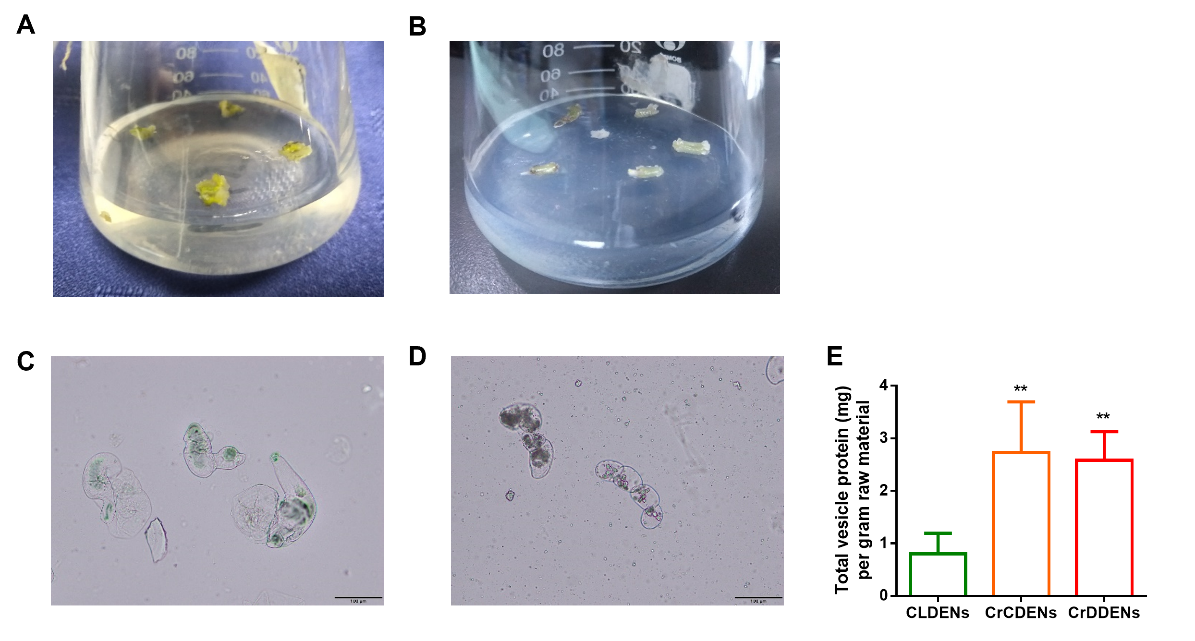


**Figure S7** Induction of *C. roseus* cells. (A) Germinating dedifferentiated cells in the leaves of *C. roseus*. (B) Germinating cambial meristematic cells in the stems of *C. roseus*. (C) *C. roseus* dedifferentiated cells viewed through a 20× optical microscope. Scale bar = 100 μm. (D) *C. roseus* cambial meristematic cells viewed through a 20× optical microscope. Scale bar = 100 μm. (E). Comparison of the yield of three nanovesicles. Data were mean ±SD, n=3, ^**^*P*<0.01 *vs.* CLDENs group.

**Table S1** List of identified substances in lipidomic analysis.

| **No.** | **Metabolite name** | **Reference *m/z*** | **Formula** |
| --- | --- | --- | --- |
| 1 | LPC O-25:0 | 608.5014 | C_33_H_70_NO_6_P |
| 2 | LPC O-26:4 | 614.45441 | C_34_H_64_NO_6_P |
| 3 | LPC O-16:4 | 474.29791 | C_24_H_44_NO_6_P |
| 4 | LPE O-10:0 | 356.2197 | C_15_H_34_NO_6_P |
| 5 | LPE O-19:3 | 474.29901 | C_24_H_46_NO_6_P |
| 6 | LPE O-12:0 | 382.23639 | C_17_H_38_NO_6_P |
| 7 | LPG O-26:1 | 607.43439 | C_32_H_65_O_8_P |
| 8 | LPG O-18:4 | 489.2623 | C_24_H_43_O_8_P |
| 9 | PC O-12:0_17:0 | 738.52911 | C_37_H_76_NO_8_P |
| 10 | PC O-8:0_18:1 | 694.46649 | C_34_H_68_NO_8_P |
| 11 | PC O-28:0_17:1 | 976.75873 | C_53_H_106_NO_9_P |
| 12 | PC O-28:0_17:2 | 974.7431 | C_53_H_104_NO_9_P |
| 13 | PC O-15:0_18:3 | 836.52948 | C_41_H_78_NO_11_P |
| 14 | PC O-18:0_22:5 | 914.61279 | C_48_H_88_NO_10_P |
| 15 | PC O-12:0_18:1 | 750.52911 | C_38_H_76_NO_8_P |
| 16 | PC O-16:0_18:2 | 852.56079 | C_42_H_82_NO_11_P |
| 17 | PC O-22:4_22:6 | 976.5921 | C_52_H_86_NO_11_P |
| 18 | PC O-18:0_22:6 | 880.6073 | C_48_H_86_NO_8_P |
| 19 | PC O-18:0_18:2 | 880.5921 | C_44_H_86_NO_11_P |
| 20 | PE O-17:2_24:0 | 814.63312 | C_46_H_90_NO_8_P |
| 21 | PE O-17:2_28:0 | 870.69568 | C_50_H_98_NO_8_P |
| 22 | PE O-22:5_24:0 | 878.66443 | C_51_H_94_NO_8_P |
| 23 | PE O-20:5_26:0 | 878.66443 | C_51_H_94_NO_8_P |
| 24 | PE O-28:0_18:1 | 902.72192 | C_51_H_102_NO_9_P |
| 25 | PE O-20:5_24:0 | 850.63312 | C_49_H_90_NO_8_P |
| 26 | PE O-18:2_28:0 | 884.71143 | C_51_H_100_NO_8_P |
| 27 | PC O-36:9 | 758.5119 | C_44_H_72_NO_7_P |
| 28 | PC O-36:10 | 756.49628 | C_44_H_70_NO_7_P |
| 29 | PC O-36:8 | 760.52759 | C_44_H_74_NO_7_P |
| 30 | PC O-31:5 | 696.49628 | C_39_H_70_NO_7_P |
| 31 | PC O-38:10 | 784.52759 | C_46_H_74_NO_7_P |
| 32 | PC O-33:5 | 724.52759 | C_41_H_74_NO_7_P |
| 33 | PC O-35:5 | 752.5589 | C_43_H_78_NO_7_P |
| 34 | PC O-39:5 | 808.62152 | C_47_H_86_NO_7_P |
| 35 | PC O-38:9 | 786.54321 | C_46_H_76_NO_7_P |
| 36 | PC O-37:5 | 780.59021 | C_45_H_82_NO_7_P |
| 37 | PC O-40:8 | 816.59021 | C_48_H_82_NO_7_P |
| 38 | PC O-19:0_24:1 | 916.73761 | C_51_H_102_NO_7_P |
| 39 | PC O-55:3 | 1036.9032 | C_63_H_122_NO_7_P |
| 40 | PC O-37:3\|PC O-19:1_18:2 | 828.61243 | C_45_H_86_NO_7_P |
| 41 | PC O-35:3\|PC O-17:0_18:3 | 800.58112 | C_43_H_82_NO_7_P |
| 42 | PC O-22:1_28:7 | 1000.73761 | C_58_H_102_NO_7_P |
| 43 | PC O-38:8 | 788.5589 | C_46_H_78_NO_7_P |
| 44 | PC O-44:7 | 874.6684 | C_52_H_92_NO_7_P |
| 45 | PC O-20:0_28:5 | 978.75317 | C_56_H_104_NO_7_P |
| 46 | PC O-33:8 | 718.48059 | C_41_H_68_NO_7_P |
| 47 | PC O-22:2_28:7 | 998.72192 | C_58_H_100_NO_7_P |
| 48 | PE O-20:0_28:6 | 888.68518 | C_53_H_96_NO_7_P |
| 49 | PE O-24:5_18:1 | 804.59131 | C_47_H_84_NO_7_P |
| 50 | PE O-16:0_28:1 | 842.70081 | C_49_H_98_NO_7_P |
| 51 | PE O-24:5_18:2 | 802.57562 | C_47_H_82_NO_7_P |
| 52 | PE O-22:6_16:0 | 748.52869 | C_43_H_76_NO_7_P |
| 53 | PE O-26:5_20:1 | 860.65387 | C_51_H_92_NO_7_P |
| 54 | PE O-24:5_18:3 | 800.56 | C_47_H_80_NO_7_P |
| 55 | PE O-34:5\|PE O-16:2_18:3 | 694.48169 | C_39_H_70_NO_7_P |
| 56 | PE O-19:1_18:2 | 740.56 | C_42_H_80_NO_7_P |
| 57 | PE O-24:5_16:1 | 776.56 | C_45_H_80_NO_7_P |
| 58 | PE O-26:5_18:1 | 832.62262 | C_49_H_88_NO_7_P |
| 59 | PE O-26:7_18:2 | 826.57562 | C_49_H_82_NO_7_P |
| 60 | PE O-26:6_18:1 | 830.60687 | C_49_H_86_NO_7_P |
| 61 | PE O-22:0_28:6 | 916.71649 | C_55_H_100_NO_7_P |
| 62 | PE O-22:6_16:1 | 746.513 | C_43_H_74_NO_7_P |
| 63 | PE O-18:0_28:1 | 870.73212 | C_51_H_102_NO_7_P |
| 64 | PE O-19:0_28:6 | 874.66949 | C_52_H_94_NO_7_P |
| 65 | PE P-22:5_26:7 | 876.59021 | C_53_H_82_NO_7_P |
| 66 | PE O-19:0_26:1 | 856.71649 | C_50_H_100_NO_7_P |
| 67 | PG O-13:0_28:7 | 819.5545 | C_47_H_81_O_9_P |
| 68 | PG O-26:7_26:7 | 959.61713 | C_58_H_89_O_9_P |
| 69 | PG O-27:1_16:0 | 859.67969 | C_49_H_97_O_9_P |
| 70 | PG O-24:6_28:7 | 961.63281 | C_58_H_91_O_9_P |
| 71 | PG O-18:4_23:1 | 823.58582 | C_47_H_85_O_9_P |
| 72 | PG O-21:1_18:3 | 797.57019 | C_45_H_83_O_9_P |
| 73 | PG O-26:1_16:0 | 845.66412 | C_48_H_95_O_9_P |
| 74 | PG O-21:1_18:2 | 799.58582 | C_45_H_85_O_9_P |
| 75 | PG O-15:4_19:5 | 717.4137 | C_40_H_63_O_9_P |
| 76 | PG O-18:2_21:4 | 793.53888 | C_45_H_79_O_9_P |
| 77 | PG O-23:1_18:3 | 825.6015 | C_47_H_87_O_9_P |
| 78 | PG O-26:7_28:7 | 987.64838 | C_60_H_93_O_9_P |
| 79 | PG O-16:1_27:1 | 857.66412 | C_49_H_95_O_9_P |
| 80 | PG O-17:0_28:4 | 881.66412 | C_51_H_95_O_9_P |
| 81 | PG O-17:0_28:3 | 883.67969 | C_51_H_97_O_9_P |
| 82 | PG O-21:3_18:2 | 795.5545 | C_45_H_81_O_9_P |
| 83 | PG O-8:0_14:0 | 567.3667 | C_28_H_57_O_9_P |
| 84 | PG O-16:1_13:1 | 661.44501 | C_35_H_67_O_9_P |
| 85 | PG O-22:4_28:7 | 937.63281 | C_56_H_91_O_9_P |
| 86 | PG O-27:1_18:1 | 885.69537 | C_51_H_99_O_9_P |
| 87 | PG O-8:0_16:0 | 595.39801 | C_30_H_61_O_9_P |
| 88 | PG O-8:0_19:4 | 629.38239 | C_33_H_59_O_9_P |
| 89 | PG O-17:3_28:7 | 869.57019 | C_51_H_83_O_9_P |
| 90 | PG O-17:0_28:2 | 885.69537 | C_51_H_99_O_9_P |
| 91 | PG O-23:1_18:2 | 827.61713 | C_47_H_89_O_9_P |
| 92 | PG O-27:1_16:1 | 857.66412 | C_49_H_95_O_9_P |
| 93 | PG O-28:0_28:7 | 1029.78931 | C_62_H_111_O_9_P |
| 94 | PI O-8:0_26:1 | 821.55487 | C_43_H_83_O_12_P |
| 95 | PI O-17:2_28:7 | 959.60193 | C_54_H_89_O_12_P |
| 96 | PI O-23:1_22:6 | 963.63318 | C_54_H_93_O_12_P |
| 97 | PI O-8:0_26:4 | 815.508 | C_43_H_77_O_12_P |
| 98 | PI O-23:1_20:5 | 937.61749 | C_52_H_91_O_12_P |
| 99 | PI O-23:0_18:3 | 915.63318 | C_50_H_93_O_12_P |
| 100 | PI O-23:1_18:3 | 913.61749 | C_50_H_91_O_12_P |
| 101 | PI O-13:0_28:4 | 913.61749 | C_50_H_91_O_12_P |
| 102 | PI O-23:1_18:2 | 915.63318 | C_50_H_93_O_12_P |
| 103 | PI O-8:0_26:3 | 817.52362 | C_43_H_79_O_12_P |
| 104 | PI O-21:0_28:1 | 1031.78967 | C_58_H_113_O_12_P |
| 105 | PI O-8:0_24:1 | 793.52362 | C_41_H_79_O_12_P |
| 106 | PI O-15:0_28:7 | 935.60193 | C_52_H_89_O_12_P |
| 107 | PI O-13:0_28:3 | 915.63318 | C_50_H_93_O_12_P |
| 108 | PC 17:2_28:0 | 972.72742 | C_53_H_102_NO_9_P |
| 109 | PE 10:0_22:6 | 710.40387 | C_37_H_62_NO_10_P |
| 110 | PG 17:0_18:1 | 809.51862 | C_41_H_79_O_13_P |
| 111 | PG 20:3_18:2 | 811.51312 | C_44_H_77_O_11_P |
| 112 | PG 16:0_18:0 | 765.52869 | C_40_H_79_O_11_P |
| 113 | PG 14:1_4:0 | 539.2627 | C_24_H_45_O_11_P |
| 114 | PI 22:3_22:3 | 981.60742 | C_53_H_91_O_14_P |
| 115 | PI 20:5_24:0 | 983.62299 | C_53_H_93_O_14_P |
| 116 | PI 20:4_24:0 | 985.63867 | C_53_H_95_O_14_P |
| 117 | PS 24:0_26:0 | 1002.77441 | C_56_H_110_NO_11_P |
| 118 | PA 34:1\|PA 16:0_18:1 | 673.48138 | C_37_H_71_O_8_P |
| 119 | PA 34:2\|PA 16:0_18:2 | 671.4657 | C_37_H_69_O_8_P |
| 120 | PA 22:0_8:0 | 619.43439 | C_33_H_65_O_8_P |
| 121 | PA 36:3\|PA 18:1_18:2 | 697.48138 | C_39_H_71_O_8_P |
| 122 | PA 36:4\|PA 18:2_18:2 | 695.4657 | C_39_H_69_O_8_P |
| 123 | PA 13:0_28:2 | 769.57532 | C_44_H_83_O_8_P |
| 124 | PA 17:3_28:7 | 809.5127 | C_48_H_75_O_8_P |
| 125 | PA 13:0_28:3 | 767.55957 | C_44_H_81_O_8_P |
| 126 | PA 23:1_18:2 | 767.55957 | C_44_H_81_O_8_P |
| 127 | PA 18:3_18:3 | 691.43439 | C_39_H_65_O_8_P |
| 128 | PA 9:0_24:3 | 655.43439 | C_36_H_65_O_8_P |
| 129 | PC 6:0_38:3 | 896.71033 | C_52_H_98_NO_8_P |
| 130 | PC 19:0_23:1 | 872.71033 | C_50_H_98_NO_8_P |
| 131 | PC 6:0_38:4 | 894.69458 | C_52_H_96_NO_8_P |
| 132 | PC 22:1_22:1 | 898.72589 | C_52_H_100_NO_8_P |
| 133 | PC 34:2\|PC 16:0_18:2 | 802.56042 | C_42_H_80_NO_8_P |
| 134 | PC 21:1_21:1 | 870.69458 | C_50_H_96_NO_8_P |
| 135 | PC 6:0_38:5 | 892.67902 | C_52_H_94_NO_8_P |
| 136 | PC 6:0_36:3 | 868.67902 | C_50_H_94_NO_8_P |
| 137 | PC 6:0_24:5 | 696.4599 | C_38_H_66_NO_8_P |
| 138 | PC 36:3\|PC 18:1_18:2 | 828.57599 | C_44_H_82_NO_8_P |
| 139 | PC 76:19 | 1334.94263 | C_84_H_130_NO_8_P |
| 140 | PC 34:0_6:0 | 846.69458 | C_48_H_96_NO_8_P |
| 141 | PC 36:1\|PC 18:0_18:1 | 832.6073 | C_44_H_86_NO_8_P |
| 142 | PC 34:7_38:10 | 1260.92944 | C_80_H_126_NO_8_P |
| 143 | PC 13:0_38:9 | 982.72589 | C_59_H_100_NO_8_P |
| 144 | PC 53:9 | 1032.73926 | C_61_H_104_NO_8_P |
| 145 | PC 36:2\|PC 18:1_18:1 | 830.59167 | C_44_H_84_NO_8_P |
| 146 | PC 19:4_38:10 | 1056.74158 | C_65_H_102_NO_8_P |
| 147 | PC 6:0_38:1 | 900.74158 | C_52_H_102_NO_8_P |
| 148 | PC 58:14 | 1092.73926 | C_66_H_104_NO_8_P |
| 149 | PC 37:7 | 812.52008 | C_45_H_76_NO_8_P |
| 150 | PC 36:4\|PC 18:2_18:2 | 826.56042 | C_44_H_80_NO_8_P |
| 151 | PC 19:0_19:0 | 818.66333 | C_46_H_92_NO_8_P |
| 152 | PC 55:11 | 1056.73926 | C_63_H_104_NO_8_P |
| 153 | PC 16:4_32:9 | 976.6073 | C_56_H_86_NO_8_P |
| 154 | PC 6:0_20:1 | 648.4599 | C_34_H_66_NO_8_P |
| 155 | PC 62:17 | 1142.75476 | C_70_H_106_NO_8_P |
| 156 | PC 74:12 | 1321.02087 | C_82_H_140_NO_8_P |
| 157 | PE 45:12 | 872.52008 | C_50_H_76_NO_8_P |
| 158 | PE 6:0_31:1 | 758.5705 | C_42_H_82_NO_8_P |
| 159 | PE 22:4_36:9 | 1030.72595 | C_63_H_100_NO_8_P |
| 160 | PE 36:2\|PE 18:1_18:1 | 742.53918 | C_41_H_78_NO_8_P |
| 161 | PE 18:3_18:3 | 736.49121 | C_41_H_70_NO_8_P |
| 162 | PE 34:2\|PE 16:0_18:2 | 714.50787 | C_39_H_74_NO_8_P |
| 163 | PE 36:3 | 742.53809 | C_41_H_76_NO_8_P |
| 164 | PE 20:1_18:2 | 770.5694 | C_43_H_80_NO_8_P |
| 165 | PE 34:1\|PE 16:0_18:1 | 716.52362 | C_39_H_76_NO_8_P |
| 166 | PE 36:5\|PE 18:2_18:3 | 736.49231 | C_41_H_72_NO_8_P |
| 167 | PE 74:15 | 1272.927 | C_79_H_128_NO_8_P |
| 168 | PE 36:4\|PE 18:2_18:2 | 738.50787 | C_41_H_74_NO_8_P |
| 169 | PE 9:0_38:1 | 898.72699 | C_52_H_102_NO_8_P |
| 170 | PE 15:0_38:4 | 976.77399 | C_58_H_108_NO_8_P |
| 171 | PG 7:0_24:2 | 722.4967 | C_37_H_69_O_10_P |
| 172 | PG 19:3_38:10 | 1064.73145 | C_63_H_99_O_10_P |
| 173 | PG 6:0_17:3 | 608.35577 | C_29_H_51_O_10_P |
| 174 | PG 32:1\|PG 16:0_16:1 | 719.48688 | C_38_H_73_O_10_P |
| 175 | PG 24:0_6:0 | 693.47119 | C_36_H_71_O_10_P |
| 176 | PG 16:1_38:10 | 1026.71582 | C_60_H_97_O_10_P |
| 177 | PG 6:0_13:1 | 537.28339 | C_25_H_47_O_10_P |
| 178 | PG 6:0_26:5 | 711.42432 | C_38_H_65_O_10_P |
| 179 | PG 34:1\|PG 16:0_18:1 | 747.51819 | C_40_H_77_O_10_P |
| 180 | PG 34:2\|PG 16:0_18:2 | 745.5025 | C_40_H_75_O_10_P |
| 181 | PG 16:3_38:10 | 1022.68451 | C_60_H_93_O_10_P |
| 182 | PG 19:0_38:7 | 1057.78418 | C_63_H_111_O_10_P |
| 183 | PG 27:0_38:9 | 1184.91919 | C_71_H_123_O_10_P |
| 184 | PG 15:0_18:1(d7) | 740.54657 | C_39_H_68_D_7_O_10_P |
| 185 | PG 6:0_24:1 | 691.45563 | C_36_H_69_O_10_P |
| 186 | PG 21:0_38:4 | 1110.90356 | C_65_H_121_O_10_P |
| 187 | PG 7:0_22:6 | 686.40277 | C_35_H_57_O_10_P |
| 188 | PG 6:0_30:1 | 775.5495 | C_42_H_81_O_10_P |
| 189 | PG 21:0_38:10 | 1079.76855 | C_65_H_109_O_10_P |
| 190 | PG 7:0_36:2 | 890.68451 | C_49_H_93_O_10_P |
| 191 | PG 30:0_6:0 | 777.56512 | C_42_H_83_O_10_P |
| 192 | PG 6:0_21:3 | 664.4184 | C_33_H_59_O_10_P |
| 193 | PG 16:3_34:9 | 949.59637 | C_56_H_87_O_10_P |
| 194 | PG 7:0_34:6 | 835.5495 | C_47_H_81_O_10_P |
| 195 | PG 6:0_20:5 | 627.33038 | C_32_H_53_O_10_P |
| 196 | PG 32:9_32:9 | 1152.7627 | C_70_H_103_O_10_P |
| 197 | PG 15:3_34:9 | 935.58081 | C_55_H_85_O_10_P |
| 198 | PG 15:3_15:4 | 679.36169 | C_36_H_57_O_10_P |
| 199 | PI 35:2 | 871.5307 | C_44_H_81_O_13_P |
| 200 | PI 34:6 | 849.45239 | C_43_H_71_O_13_P |
| 201 | PI 70:19 | 1327.81238 | C_79_H_117_O_13_P |
| 202 | PI 20:0 | 660.37189 | C_29_H_55_O_13_P |
| 203 | PI 62:17 | 1214.76306 | C_71_H_105_O_13_P |
| 204 | PI 63:4 | 1259.93762 | C_72_H_133_O_13_P |
| 205 | PI 45:3 | 1004.71619 | C_54_H_99_O_13_P |
| 206 | PI 48:11 | 1035.59326 | C_57_H_89_O_13_P |
| 207 | PI 12:0_38:2 | 1057.76904 | C_59_H_111_O_13_P |
| 208 | PI 67:8 | 1307.93762 | C_76_H_133_O_13_P |
| 209 | PI 61:3 | 1233.922 | C_70_H_131_O_13_P |
| 210 | PI 40:11 | 923.46808 | C_49_H_73_O_13_P |
| 211 | PI 49:2 | 1067.74976 | C_58_H_109_O_13_P |
| 212 | PI 73:7 | 1394.04724 | C_82_H_147_O_13_P |
| 213 | PI 61:2 | 1235.93762 | C_70_H_133_O_13_P |
| 214 | PI 35:8 | 859.43683 | C_44_H_69_O_13_P |
| 215 | PI 48:1 | 1055.74976 | C_57_H_109_O_13_P |
| 216 | PI 36:7 | 875.46808 | C_45_H_73_O_13_P |
| 217 | PI 7:0_36:7 | 949.58118 | C_52_H_87_O_13_P |
| 218 | PI 12:0 | 553.20203 | C_21_H_39_O_13_P |
| 219 | PI 58:13 | 1166.76306 | C_67_H_105_O_13_P |
| 220 | PI 42:9 | 955.5307 | C_51_H_81_O_13_P |
| 221 | PI 14:0_38:4 | 1081.76904 | C_61_H_111_O_13_P |
| 222 | PI 38:7 | 903.49939 | C_47_H_77_O_13_P |
| 223 | PI 21:1 | 677.32721 | C_30_H_55_O_13_P |
| 224 | PI 7:0_38:10 | 971.56549 | C_54_H_85_O_13_P |
| 225 | PI 14:0_38:3 | 1083.78455 | C_61_H_113_O_13_P |
| 226 | PI 12:0_38:1 | 1059.78455 | C_59_H_113_O_13_P |
| 227 | PI 43:0 | 987.68719 | C_52_H_101_O_13_P |
| 228 | PI 70:5 | 1356.03149 | C_79_H_145_O_13_P |
| 229 | PI 22:1 | 686.38751 | C_31_H_57_O_13_P |
| 230 | PS 69:9 | 1258.9325 | C_75_H_130_NO_10_P |
| 231 | PS 63:3 | 1186.9325 | C_69_H_130_NO_10_P |
| 232 | PS 67:2 | 1245.01074 | C_73_H_140_NO_10_P |

**Table S2** List of identified substances in metabolomic analysis.

| **No.** | **Metabolite name** | **Reference *m/z*** | **Formula** |
| --- | --- | --- | --- |
|  |  |  |  |
| 1 | (1R,2R,4S,7R,8S,12R)-7-(furan-3-yl)-1,8,12,17,17-pentamethyl-3,6,16-trioxapentacyclo [9.9.02,4.02,8.012,18] icos-13-ene-5,15,20-trione | 453.19189 | C_26_H_30_O_7_ |
| 2 | (1S,8R,9R)-8-hydroxy-4-(propan-2-ylidene)-10-oxatricyclo [7.2.1.01,5] dodecane-8-carboxylic acid | 265.14453 | C_15_H_22_O_4_ |
| 3 | (2R)-5-methoxy-2-methyl-2,3,8,9-tetrahydrofuro [2,3-h] chromen-4-one | 235.09621 | C_13_H_14_O_4_ |
| 4 | (2R,3R,4S,6S)-6-(((2R,3S,4S,6S)-6-(((2R,3S,4S,6R)-6-(((10S,13S)-12,14-dihydroxy-10,13-dimethyl-17-(5-oxo-2,5-dihydrofuran-3-yl) hexadecahydro-1H-cyclopenta[a]phenanthren-3-yl) oxy)-4-hydroxy-2-methyltetrahydro-2H-pyran-3-yl) oxy)-4-hydroxy-2-methyltetrahydro-2H-pyran-3-yl) oxy)-2-methyl-3-(((2S,3R,4S,5S,6R)-3,4,5-trihydroxy-6-(hydroxymethyl) tetrahydro-2H-pyran-2-yl)oxy)tetrahydro-2H-pyran-4-yl acetate | 985.5 | C_49_H_76_O_20_ |
| 5 | (2S)-6-[(2S)-5,7-dihydroxy-2-(4-hydroxyphenyl)-4-oxo-2,3-dihydrochromen-8-yl]-5,7-dihydroxy-2-(4-hydroxyphenyl)-2,3-dihydrochromen-4-one | 543.12738 | C_30_H_22_O_10_ |
| 6 | (2S,3R)-2-(((S)-7-acetamido-1,2,3-trimethoxy-9-oxo-5,6,7,9-tetrahydrobenzo[a]heptalen-10-yl) amino)-N-(1-benzylpiperidin-4-yl)-3-methylpentanamide | 693.35999 | C_39_H_50_N_4_O_6_ |
| 7 | (2S,3S,4S,5R,6S)-6-[2-(3,4-dihydroxyphenyl)-5-hydroxy-4-oxochromen-7-yl]oxy-3,4,5-trihydroxyoxane-2-carboxylic acid | 461.0726 | C_21_H_18_O_12_ |
| 8 | (3,9,16)-20-Hydroxy-16,23:16,30-diepoxydammar-24-en-3-yl 6-deoxy-L-mannopyranosyl-(1->2)-[D-glucopyranosyl-(1->6)-D-glucopyranosyl-(1->3)]-L-arabinopyranoside | 1113.52417 | C_53_H_86_O_22_ |
| 9 | (3a1R,4R,5S,5aR,10bR)-methyl 4-acetoxy-3a-ethyl-5-hydroxy-8-methoxy-6-methyl-3a,3a1,4,5,5a,6,11,12-octahydro-1H-indolizino[8,1-cd] carbazole-5-carboxylate | 457.23001 | C_25_H_32_N_2_O_6_ |
| 10 | (3aR,3a1S,5S,5aS,10bS)-methyl 6-acetyl-3a-ethyl-2,3,3a,3a1,4,5,5a,6,11,12-decahydro-1H-indolizino[8,1-cd] carbazole-5-carboxylate | 383.23001 | C_23_H_30_N_2_O_3_ |
| 11 | (3R,4S)-4,6,8-trihydroxy-7-methoxy-3-methyl-3,4-dihydroisochromen-1-one | 239.05679 | C_11_H_12_O_6_ |
| 12 | (E)-(3S,10R,13R)-10,13-dimethyl-17-(6-methylheptan-2-yl)-2,3,4,7,8,9,10,11,12,13,14,15,16,17-tetradecahydro-1H-cyclopenta[a]phenanthren-3-yl 3-chlorobut-2-enoate | 511.32999 | C_31_H_49_ClO_2_ |
| 13 | (R)-4-aminoisoxazolidin-3-one | 103.05 | C_3_H_6_N_2_O_2_ |
| 14 | [(2S,3R,4R,5S,6S)-2-[2-(3,4-dihydroxyphenyl)-5,7-dihydroxy-4-oxochromen-3-yl] oxy-3,5-dihydroxy-6-methyloxan-4-yl] 3,4,5-trihydroxybenzoate | 599.10419 | C_28_H_24_O_15_ |
| 15 | 1-hydroxyanthraquinone | 223.04007 | C_14_H_8_O_3_ |
| 16 | 2-(2-hydroxy-6-methylhept-5-en-2-yl)-7-(3-methylbut-2-enyl)-2,3-dihydro-1-benzofuran-5-carboxylic acid | 357.20639 | C_22_H_30_O_4_ |
| 17 | 2-(2-hydroxy-6-methylhept-5-en-2-yl)-7-(3-methylbut-2-enyl)-2,3-dihydro-1-benzofuran-5-carboxylic acid | 357.20639 | C_22_H_30_O_4_ |
| 18 | 2-Acetylacteoside | 689.20001 | C_31_H_38_O_16_ |
| 19 | 2-Benzyl-4-chlorophenol | 217.04257 | C_13_H_11_ClO |
| 20 | 2-Hydroxybenzaldehyde | 121.0295 | C_7_H_6_O_2_ |
| 21 | 2-morpholino-7H-naphtho[1,2,3-de] quinolin-7-one | 317.13 | C_20_H_16_N_2_O_2_ |
| 22 | 3-(4-methoxy-phenyl)-isochromen-1-one | 275.07001 | C_16_H_12_O_3_ |
| 23 | 3,3'-(6,7,9,10,17,18,20,21-octahydrodibenzo [b, k] [1,4,7,10,13,16] hexaoxacyclooctadecine-2,13-dicarbonyl) bis(3,4,5,6-tetrahydro-1H-1,5-methanopyrido[1,2-a] [1,5] diazocin-8(2H)-one) | 815.33002 | C_44_H_48_N_4_O_10_ |
| 24 | 4-((3S,5S,10R,13R,14S,17S)-3-(((2R,4S,5S,6R)-4,5-dihydroxy-6-methyltetrahydro-2H-pyran-2-yl) oxy)-5,14-dihydroxy-10-(hydroxymethyl)-13-methylhexadecahydro-1H-cyclopenta[a]phenanthren-17-yl) furan-2(5H)-one | 559.28003 | C_29_H_44_O_9_ |
| 25 | 4-(1H-indol-3-yl) butan-2-one | 188.11 | C_12_H_13_NO |
| 26 | 4-(sec-butoxy) benzoic acid | 217.08 | C_11_H_14_O_3_ |
| 27 | 4-hydroxy-3-[(2E)-4-hydroxy-3,7-dimethylocta-2,6-dienyl]-5-[(E)-4-hydroxy-3-methylbut-2-enyl] benzoic acid | 397.1987 | C_22_H_30_O_5_ |
| 28 | 5-hydroxy-2,2,6,6-tetramethyl-4-[2-methyl-1-[2,4,6-trihydroxy-3-(2-methylpropanoyl) phenyl] propyl] cyclohex-4-ene-1,3-dione | 431.2077 | C_24_H_32_O_7_ |
| 29 | 5-Methoxypsoralen | 215.03499 | C_12_H_8_O_4_ |
| 30 | 6-[[(3S,6aR,6bS,8aS,14bR)-4,4,6a,6b,11,11,14b-heptamethyl-8a-[3,4,5-trihydroxy-6-(hydroxymethyl)oxan-2-yl]oxycarbonyl-1,2,3,4a,5,6,7,8,9,10,12,12a,14,14a- tetradecahydropicen-3-yl]oxy]-3,5-dihydroxy-4-[3,4,5-trihydroxy-6-(hydroxymethyl) oxan-2-yl] oxyoxane-2-carboxylic acid | 955.49078 | C_48_H_76_O_19_ |
| 31 | 7-hydroxy-2-(4-hydroxy-3,5-dimethoxyphenyl)-5-[(2S,3R,4S,5S,6R)-3,4,5-trihydroxy-6-(hydroxymethyl) oxan-2-yl] oxychromen-4-one | 491.11951 | C_23_H_24_O_12_ |
| 32 | alpha-L-(-)-Fucose 1-phosphate bis(cyclohexylammonium) salt | 243.02753 | C_6_H_13_O_8_P |
| 33 | Arnicolide D | 355.14999 | C_19_H_24_O_5_ |
| 34 | Azelaic acid | 187.09758 | C_9_H_16_O_4_ |
| 35 | Butylparaben | 193.08702 | C_11_H_14_O_3_ |
| 36 | Caffeic acid | 179.03499 | C_9_H_8_O_4_ |
| 37 | Cimiracemoside D | 679.40002 | C_37_H_58_O_11_ |
| 38 | Coniferyl alcohol + O-Hex | 387.12909 | C_16_H_22_O_8_ |
| 39 | D-(+)-Melezitose | 503.16177 | C_18_H_32_O_16_ |
| 40 | Deferrioxamine E | 599.341 | C_27_H_48_N_6_O_9_ |
| 41 | Demethyleneberberine | 347.10999 | C_19_H_18_NO_4_^+^ |
| 42 | DErySphingosine | 300.2897 | C_18_H_37_NO_2_ |
| 43 | DGMG 18:3 | 721.36292 | C_33_H_56_O_14_ |
| 44 | Emodin 8-glucoside | 455.10001 | C_21_H_20_O_10_ |
| 45 | Euphodendroidin K | 793.34698 | C_41_H_54_O_14_ |
| 46 | FA 18:1+1O | 297.24399 | C_18_H_34_O_3_ |
| 47 | FA 18:1+3O | 329.2312 | C_18_H_34_O_5_ |
| 48 | FA 18:2+1O | 295.22739 | C_18_H_32_O_3_ |
| 49 | FA 18:2+2O | 311.22159 | C_18_H_32_O_4_ |
| 50 | FA 18:2+3O | 327.21631 | C_18_H_32_O_5_ |
| 51 | FA 18:2+O | 293.21231 | C_18_H_30_O_3_ |
| 52 | FA 18:3+1O | 293.21161 | C_18_H_30_O_3_ |
| 53 | FA 18:4+1O | 291.19449 | C_18_H_28_O_3_ |
| 54 | FA 18:4+2O | 309.20749 | C_18_H_30_O_4_ |
| 55 | Fisetin | 285.04099 | C_15_H_10_O_6_ |
| 56 | gamma-Glutamyltyrosine | 309.10922 | C_14_H_18_N_2_O_6_ |
| 57 | Gardenin B | 381.095 | C_19_H_18_O_7_ |
| 58 | Glycocholic Acid | 488.29825 | C_26_H_43_NO_6_ |
| 59 | Gly-Leu | 187.10881 | C_8_H_16_N_2_O_3_ |
| 60 | Harmine hydrochloride | 249.08 | C_13_H_13_ClN_2_O |
| 61 | Higenamine | 272.12 | C_16_H_17_NO_3_ |
| 62 | Hydroxyferulic acid | 209.04469 | C_10_H_10_O_5_ |
| 63 | Hymenamide D | 792.396 | C_38_H_55_N_7_O_10_ |
| 64 | Imazamox | 304.13028 | C_15_H_19_N_3_O_4_ |
| 65 | Indigo Carmine | 263.08151 | C_16_H_10_N_2_O_2_ |
| 66 | Isoleucylisoleucine | 243.1716 | C_12_H_24_N_2_O_3_ |
| 67 | Koumidine | 293.16592 | C_19_H_22_N_2_O |
| 68 | Koumidine | 293.16592 | C_19_H_22_N_2_O |
| 69 | L-(-)-Phenylalanine | 164.0717 | C_9_H_11_NO_2_ |
| 70 | LPC 16:0 | 540.33069 | C_24_H_50_NO_7_P |
| 71 | LPC 18:1 | 566.34521 | C_26_H_52_NO_7_P |
| 72 | LPC 18:2 | 564.32727 | C_26_H_50_NO_7_P |
| 73 | Maltotriose | 527.15826 | C_18_H_32_O_16_ |
| 74 | methyl asterrate | 361.0928 | C_18_H_18_O_8_ |
| 75 | Methyl gallate | 185.03999 | C_8_H_8_O_5_ |
| 76 | Methylophiopogonanone A | 365.10001 | C_19_H_18_O_6_ |
| 77 | MGMG 18:3 | 559.31073 | C_27_H_46_O_9_ |
| 78 | Monolinolein | 393.24014 | C_21_H_38_O_4_ |
| 79 | Myricetin | 317.0303 | C_15_H_10_O_8_ |
| 80 | Oleic acid | 281.2486 | C_18_H_34_O_2_ |
| 81 | Palmitic Acid | 255.233 | C_16_H_32_O_2_ |
| 82 | Phytosphingosine | 318.29941 | C_18_H_39_NO_3_ |
| 83 | Piperazine-2,5-dione | 115.05 | C_4_H_6_N_2_O_2_ |
| 84 | Prosapogenin A | 745.40997 | C_39_H_62_O_12_ |
| 85 | Pyrenophorol | 311.19281 | C_16_H_24_O_6_ |
| 86 | Quercetin | 301.03537 | C_15_H_10_O_7_ |
| 87 | Serpentine | 349.155 | [C_21_H_21_N_2_O_3_]^+^ |
| 88 | Soyasaponin Ba | 957.50647 | C_48_H_78_O_19_ |
| 89 | Stearic acid | 283.26425 | C_18_H_36_O_2_ |
| 90 | Sucrose | 341.10895 | C_12_H_22_O_11_ |
| 91 | Sulfadimethoxine | 311.08084 | C_12_H_14_N_4_O_4_S |
| 92 | Syringetin-3-O-glucoside | 509.12897 | C_23_H_24_O_13_ |
| 93 | Tabersonine | 337.19104 | C_21_H_24_N_2_O_2_ |
| 94 | Tenuifoliside A | 705.20001 | C_31_H_38_O_17_ |
| 95 | Tetrasaccharides (Hex-Hex-Hex-Hex) | 711.21698 | C_24_H_42_O_21_ |
| 96 | Theophylline | 203.05 | C_7_H_8_N_4_O_2_ |
| 97 | Threonine | 120.06552 | C_4_H_9_NO_3_ |
| 98 | Trehalose | 341.10889 | C_12_H_22_O_11_ |
| 99 | Trifluoroacetic acid | 112.98559 | C_2_HF_3_O_2_ |
| 100 | Tryptophan | 203.0826 | C_11_H_12_N_2_O_2_ |
| 101 | Tryptophanol | 144.082 | C_10_H_11_NO |
| 102 | Valsartan | 434.21976 | C_24_H_29_N_5_O_3_ |
| 103 | Vinpocetine | 373.19 | C_22_H_26_N_2_O_2_ |

**Table S3** List of disease phenotypes that correlated with CLDENs.

| **No.** | **Disease Phenotype** | ***p*-value** |
| --- | --- | --- |
| 1 | Renal tubular dysgenesis | *p*＜0.001 |
| 2 | Essential hypertension | *p*＜0.001 |
| 3 | Obesity leanness | *p*＜0.01 |
| 4 | Glycine encephalopathy | *p*＜0.01 |
| 5 | Maple syrup urine disease | *p*＜0.01 |
| 6 | Obsessive-compulsive disorder | *p*＜0.01 |
| 7 | Paraganglioma and gastric stromal sarcoma | *p*＜0.01 |
| 8 | Alcohol dependence | *p*＜0.01 |
| 9 | Epithelial ovarian cancer | *p*＜0.01 |
| 10 | Congenital central hypoventilation syndrome | *p*＜0.01 |
| 11 | Breast cancer | *p*＜0.01 |
| 12 | Permanent neonatal diabetes mellitus | *p*＜0.01 |
| 13 | Tobacco addiction, susceptibility | *p*＜0.01 |
| 14 | Migraine with or without aura, susceptibility | *p*＜0.01 |
| 15 | Gastrointestinal stromal tumor | *p*＜0.01 |
| 16 | Folate-sensitive neural tube defects | *p*＜0.01 |
| 17 | Amyotrophic lateral sclerosis 1 | *p*＜0.01 |
| 18 | Asthma, susceptibility | *p*＜0.01 |
| 19 | Ischemic stroke | *p*＜0.01 |
| 20 | Attention deficit-hyperactivity disorder | *p*＜0.01 |
| 21 | Albinism, oculocutaneous, Type Ⅱ | *p*＜0.01 |
| 22 | Combined cellular and humoral immune defects with granulomas | *p*＜0.01 |
| 23 | Severe combined immunodeficiency, autosomal recessive, T Cell-Negative, B Cell-Negative, NK Cell-Positive | *p*＜0.01 |
| 24 | Dravet syndrome | *p*＜0.01 |
| 25 | Hyperbilirubinemia, rotor type | *p*＜0.01 |
| 26 | Human immunodeficiency virus, Type Ⅰ, susceptibility | *p*＜0.05 |
| 27 | Schizophrenia | *p*＜0.05 |
| 28 | Brachydactyly, Type A2 | *p*＜0.05 |
| 29 | Cystic fibrosis | *p*＜0.05 |
| 30 | Ehlers-Danlos syndrome | *p*＜0.05 |
| 31 | Amyloidosis, familial visceral | *p*＜0.05 |
| 32 | Lacrimo-auriculo-dento-digital syndrome | *p*＜0.05 |
| 33 | Renal adysplasia | *p*＜0.05 |
| 34 | Omenn syndrome | *p*＜0.05 |
| 35 | Familial-hypercholesterolemia | *p*＜0.05 |

**Table S4** Proteins significantly up-regulated in differential proteomics (Top 20).

| **Protein description** | **CLDENs/PLANT Ratio** | **MW [kDa]** | **Subcellular localization** |
| --- | --- | --- | --- |
| Pyruvate decarboxylase | 195.374 | 63.282 | chloroplast |
| ABC transporter C family member 10 | 189.247 | 164.92 | plasma membrane |
| Probable metal-nicotianamine transporter YSL6 isoform X1 | 169.28 | 73.899 | plasma membrane |
| Glycerophosphodiester phosphodiesterase | 112.161 | 82.366 | plasma membrane |
| DnaJ homolog subfamily C GRV2-like | 105.353 | 284.7 | cytoplasm |
| ABC transporter C family member 4-like | 100.557 | 97.526 | plasma membrane |
| AP-3 complex subunit delta | 73.599 | 95.712 | cytoplasm |
| Ferritin | 67.649 | 23.092 | cytoplasm |
| Pyruvate decarboxylase | 57.098 | 65.065 | cytoplasm |
| Ferritin | 56.883 | 29.384 | chloroplast |
| ABC transporter C family member 2 | 56.078 | 93.882 | plasma membrane |
| Flotillin-like | 55.998 | 52.767 | mitochondria |
| CBS domain-containing protein CBSX6-like | 51.372 | 44.666 | mitochondria |
| Protein DETOXIFICATION | 50.706 | 59.644 | plasma membrane |
| Delta (24)-sterol reductase | 46.535 | 66.262 | cytoplasm |
| Tripeptidyl-peptidase II | 46.082 | 151.72 | chloroplast |
| H (+)-exporting diphosphatase | 45.193 | 17.988 | vacuolar membrane |
| Phosphoinositide phosphatase SAC3-like | 44.619 | 97.746 | nucleus |
| Chlorophyllase | 41.402 | 32.541 | cytoplasm |
| XK-related protein 2 | 39.938 | 28.71 | plasma membrane |

**Table S5** Real-time quantitative PCR primer sequence.

| **Gene** | **Primer Sequence (5' to3')** | |
| --- | --- | --- |
| GAPDH | Forward: | GTCGTGGAGTCTACTGGTGT |
|  | Reverse: | TGCTGACAATCTTGAGTGAG |
| PU.1 | Forward: | AAAATCAGGAACTTGTGCTGGC |
|  | Reverse: | GCCGAGAGAGAAAGGTGTCG |
| GATA1 | Forward: | TATGGCAAGACGGCACTCTAC |
|  | Reverse: | GGTGTCCAAGAACGTGTTGTT |
| CD86 | Forward: | GAATGCCAAGTACCTGGGCC |
|  | Reverse: | GGAGGATAATTGATCCTGTGGGTGG |
| CD206 | Forward: | GCT TCATCTTCGGGCCTTTG |
|  | Reverse: | AGCCCTTGGGTTGAGGATCC |
| iNOS | Forward: | CCCCGCTACTACTCCATCAG |
|  | Reverse: | CCACTGACACTTCGCACAAA |
| TNF-α | Forward: | CGGCAAACATGACTTCAGGC |
|  | Reverse: | GCACATCAAAGCGGCCATAG |
| IL-6 | Forward: | GGGACTGATGC TGGTGACAAC |
|  | Reverse: | AGCCTCCGACTTGTGAAGTGG |
| ARG-1 | Forward: | TACAAGACAGGGCTCCTTTCAG |
|  | Reverse: | TGAGTTCCGAAGCAAGCCAA |
